# Medium-Term Effects of a Tailored Web-Based Parenting Intervention to Reduce Adolescent Risk of Depression and Anxiety: 12-Month Findings From a Randomized Controlled Trial

**DOI:** 10.2196/13628

**Published:** 2019-08-15

**Authors:** Marie Bee Hui Yap, Mairead C Cardamone-Breen, Ronald M Rapee, Katherine A Lawrence, Andrew J Mackinnon, Shireen Mahtani, Anthony F Jorm

**Affiliations:** 1 School of Psychological Sciences and Turner Institute for Brain and Mental Health Monash University Melbourne Australia; 2 Melbourne School of Population and Global Health University of Melbourne Melbourne Australia; 3 Centre for Emotional Health, Macquarie University Sydney Australia; 4 Black Dog Institute, University of New South Wales Sydney Australia

**Keywords:** family, parenting, mental health, depression, anxiety, adolescent, internet, randomized controlled trial, preventive health services

## Abstract

**Background:**

Prevention of depression and anxiety disorders early in life is a global health priority. Evidence on risk and protective factors for youth internalizing disorders indicates that the family represents a strategic setting to target preventive efforts. Despite this evidence base, there is a lack of accessible, cost-effective preventive programs for parents of adolescents. To address this gap, we recently developed the Partners in Parenting (PiP) program—an individually tailored Web-based parenting program targeting evidence-based parenting risk and protective factors for adolescent depression and anxiety disorders. We previously reported the postintervention outcomes of a single-blinded parallel-group superiority randomized controlled trial (RCT) in which PiP was found to significantly improve self-reported parenting compared with an active-control condition (educational factsheets).

**Objective:**

This study aimed to evaluate the effects of the PiP program on parenting risk and protective factors and symptoms of adolescent depression and anxiety using data from the final assessment time point (12-month follow-up) of this RCT.

**Methods:**

Parents (n=359) and adolescents (n=332) were recruited primarily from secondary schools and completed Web-based assessments of parenting and adolescent depression and anxiety symptoms at baseline, postintervention (3 months later), and 12-month follow-up (317 parents, 287 adolescents). Parents in the PiP intervention condition received personalized feedback about their parenting and were recommended a series of up to 9 interactive modules. Control group parents received access to 5 educational factsheets about adolescent development and mental health. Both groups received a weekly 5-min phone call to encourage progress through their program.

**Results:**

Intervention group parents completed an average of 73.7% of their intended program. For the primary outcome of parent-reported parenting, the intervention group showed significantly greater improvement from baseline to 12-month follow-up compared with controls, with a medium effect size (Cohen *d*=0.51; 95% CI 0.30 to 0.72). When transformed data were used, greater reduction in parent-reported adolescent depressive symptoms was observed in the intervention group (Cohen *d*=−0.21; 95% CI −0.42 to −0.01). Mediation analyses revealed that these effects were mediated by improvements in parenting (indirect effect *b*=−0.08; 95% CI −0.16 to −0.01). No other significant intervention effects were found for adolescent-reported parenting or adolescent depression or anxiety symptoms. Both groups showed significant reductions in anxiety (both reporters) and depressive (parent reported) symptoms.

**Conclusions:**

PiP improved self-reported parenting for up to 9 months postintervention, but its effects on adolescent symptoms were less conclusive, and parent-reported changes were not perceived by adolescents. Nonetheless, given its scalability, PiP may be a useful low-cost, sustainable program to empower parents of adolescents.

**Trial Registration:**

Australian Clinical Trials Registration Number (ACTRN): 12615000328572; http://www.anzctr.org.au/ACTRN12615000328572.aspx (Archived by WebCite at http://www.webcitation.org/6qgsZ3Aqj).

## Introduction

### Background

Depression and anxiety disorders are common in young people, with lifetime prevalence rates of 18% and 38%, respectively, in adolescents aged 13 to 17 years [1]. The incidence of these disorders peaks during adolescence, and early-onset disorders tend to have a chronic and relapsing nature. In particular, these disorders forecast a cascade of deleterious long-term sequelae across multiple domains of functioning and increase suicide risk [2,3]. Moreover, a large proportion of the burden of disease from these disorders remains unavertable even with optimal treatment [4]. With emerging evidence suggesting an increase in the rates of depression and anxiety problems in children and young people internationally [5,6], there is an urgent need for effective preventive approaches to stem this global public health problem.

The family setting is a strategic target for implementing preventive approaches for adolescent depression and anxiety (also known as internalizing) disorders. As posited by interpersonal theories of developmental psychopathology, internalizing problems both result from and contribute to disruptions in developmentally salient interpersonal processes (starting with early parent-infant attachment), which in turn interfere with young people’s need for relatedness [7]. From an etiological perspective, parents have an important influence on young people’s risk for internalizing problems, in terms of both nature and nurture. Genetic research suggests that the heritability of liability to internalizing behaviors is high in 3-year-olds (76%) but reduces to 48% by the age of 12 years, whereas the shared environmental influence increases from zero at age 3 to 18% at age 12 [8]. More recently, a children-of-twins study found significant environmental transmission of anxiety from parents to their adolescents, but no evidence of significant genetic transmission [9], highlighting the influence of parental anxiety and associated parenting behaviors (eg, overprotection or overcontrol) in the etiology of adolescent internalizing problems. Meta-analyses of individual parenting behaviors associated with adolescent internalizing problems have found that parenting behaviors prospectively account for a small but significant amount of variance (1%-16%) [10,11]. Together, the evidence indicates that parenting behaviors are a promising target for the prevention of adolescent internalizing problems.

For the purpose of prevention, interventions need to target modifiable risk and protective factors [12]. A burgeoning body of literature has identified various risk and protective factors for adolescent depression and anxiety problems [13,14], including some that are potentially modifiable by parents [10,11,15]. These factors are posited to operate bidirectionally in the transactions between parent and child, especially as the child develops increasing autonomy during adolescence [16]. Factors that increase adolescents’ risk for depression and anxiety include interparental conflict, overinvolvement (including psychological control), and aversiveness (including harsh parental criticism and parent-adolescent conflict). Protective factors include parental warmth and acceptance, monitoring, and autonomy granting [10,11]. This evidence base delineates the parenting factors that should be translated into preventive interventions for parents of adolescents to reduce the societal burden of youth internalizing disorders.

For decades, preventive parenting interventions have been developed to capitalize on the influence parents have on their child’s development and adjustment based on the assumption that improving parenting will in turn yield benefits for the child’s mental health [17]. Unfortunately, the translation of research evidence into preventive parenting interventions continues to lag far behind the abovementioned evidence base, with a recent review identifying only 3 preventive parenting interventions (defined as programs where more than 50% of the intervention is delivered to the parent) targeting parents of adolescents [18]. One of these 3 interventions used a universal prevention approach (invited all parents regardless of their child’s level of risk; [19]), whereas the other two were selective prevention programs that targeted parents with an affective disorder [20] or HIV/AIDS [21]. In contrast, relatively more preventive parenting interventions have been developed and evaluated for parents of younger children [18]. Most existing interventions that are designed for parents of adolescents target behavioral problems not directly related to internalizing disorders, such as externalizing problems, substance use, and risky behaviors [17,22]. Importantly, preventive parenting interventions have demonstrated long-term benefits for child internalizing [18] and externalizing [17,22] outcomes that last up to 20 years after the intervention. Sandler et al [17] proposed a theoretical pathway by which parenting programs can have long-term benefits for child outcomes via program effects on parents. The most parsimonious program effect involves parents learning new skills from the program, and the use of these skills is maintained by positive responses from their children (eg, improved parent-child relationship).

However, the public health impact of preventive parenting interventions (regardless of child age) is limited by poor uptake and engagement [23]. In part, this is because most existing interventions are face-to-face group programs, which encounter the common barriers of stigma and practical logistics such as timing or scheduling, cost, travel, and child care [24,25]. Web-based platforms have the potential to overcome some of these barriers because of the anonymity and accessibility they afford [26]. However, a recent systematic review of technology-assisted parenting programs to prevent mental health problems in children aged 0 to 18 years [27] identified only 1 Web-based preventive parenting intervention that targets adolescent internalizing problems, known as PiP.

The PiP Web-based parenting intervention was developed to address the abovementioned gaps in preventive parenting resources [26]. It is an evidence-informed intervention that incorporates (1) developmental theory (including the developmental psychopathology framework) [28] and research into the role of parents in adolescent development and adjustment [10,15], (2) preventive medicine and public health approaches that advocate the targeting of risk and protective factors for prevention [12,29], and (3) the use of persuasive technologies to influence behavior change [30]. PiP draws its content from the parenting guidelines *How to prevent depression and clinical anxiety in your teenager: Strategies for parents* (henceforth referred to as the Guidelines) [31]. These Guidelines were the product of a rigorous research translation methodology comprising a systematic review of modifiable parental factors associated with adolescent depression and anxiety [10] and a Delphi study of international expert consensus about parenting strategies that can reduce adolescents’ risk of depression and anxiety disorders [32]. Using a consumer-engagement approach [33], the intervention was designed following the principles of Persuasive Systems Design [30] to be an interactive individually tailored program. The intervention has been evaluated in a randomized controlled trial (RCT) and found to produce significantly greater improvements in parent-reported parenting risk and protective factors for adolescent depression and anxiety (primary outcome) from baseline to postintervention (3 months later) compared with an active control condition (Cohen *d*=0.57). No significant group differences in changes over time were found for secondary outcomes of interest, including adolescent-reported parenting factors, and adolescent depression and anxiety symptoms, as reported by both parents and adolescents [34]. It is likely that changes on these secondary outcomes may emerge in the longer term, once the proximal intervention effects (eg, behavior changes in parents) have had time to influence the broader family system.

Indeed, in the broader parenting and family intervention literature, proponents of the developmental cascade model have argued that changing parent-related factors in the short term can lead to significant long-term benefits for the child through a progression of events over the course of development. For instance, an RCT of the New Beginnings Program for divorced families with children aged 9 to 12 years [35] found significant effects on parenting, parent-child relationship quality, and internalizing problems at postintervention, but these effects were not maintained at 6-month follow-up. However, significant long-term effects emerged at the 6-year [35] and 15-year [36] follow-up assessments on various functioning outcomes, including internalizing, externalizing, and substance use problems, and mediational analyses supported the cascade effects model. Similarly, an RCT of the Family Check-Up program for low-income parents of toddlers [37] found significant direct effects on maternal depression but not child internalizing problems in children aged 2 to 3 years, but an indirect effect (through reductions in maternal depression) on child internalizing symptoms emerged in middle childhood (age 7.5-8.5 years). These findings underscore the importance of long-term follow-up to examine the preventive effects of parenting interventions over the course of the child’s development.

### This Study

This study reports the findings from a medium-term (12-month) follow-up of families in this RCT. One primary outcome of interest was again parenting risk and protective factors. We hypothesized that the intervention effects observed at postintervention would be observed at the 12-month follow-up. Parent- and adolescent-reported symptoms were also examined as primary outcomes in this paper with the aim of investigating whether the intervention effects on parent-reported parenting factors would yield benefits in terms of adolescent depressive and anxiety symptoms by the 12-month follow-up. Specifically, we hypothesized that compared with the control group, the intervention group would show greater reductions in parent- and adolescent-reported symptoms from baseline to 12-month follow-up. We also hypothesized that parenting at postintervention would mediate adolescent symptoms at the 12-month follow-up, after accounting for parenting and symptom scores at baseline. Adolescent report of parenting was again examined as a secondary outcome measure. We predicted greater improvement in adolescent-reported parenting from baseline to 12-months follow-up in the intervention compared with the control group.

## Methods

### Design

This study was a parallel-group superiority RCT, with assessments conducted at baseline (preintervention), postintervention (3-months postbaseline), and 12-month follow-up (final assessment timepoint). The trial was prospectively registered with the Australian New Zealand Clinical Trials Registry (registration number ANZCTR12615000328572) and approved by the Monash University Human Research Ethics Committee (CF14/3887-2014002024). A detailed description of the methodology has been published by Yap et al [34]; however, the pertinent details will be described below.

### Sample Size

The sample size was determined based on an a priori power analysis. This indicated that a sample size of 294 parent-adolescent dyads (147 per group) was required to detect a small effect size (Cohen *d*=0.20), with an alpha level of .05, power of 0.80, and a repeated-measures design. To allow for approximately 15% attrition, we aimed to recruit 338 dyads (169 per group).

### Settings, Participants, and Eligibility Criteria

Eligible parents had an adolescent in the target age range (12-15 years at baseline), resided in Australia, had regular internet access, and an email account. Only 1 parent-adolescent dyad per family was eligible to participate. Computer and internet literacy were implicit eligibility criteria. Given the universal approach taken in this trial, no exclusion criteria were specified. Recruitment was primarily via secondary schools across Australia as well as online networks, social media, and mental health organizations (eg, *beyondblue* and Mental Health First Aid Australia). Baseline assessments were completed between August 2015 and November 2016 (when the desired sample size was reached), and 12-month follow-up data collection concluded in December 2017.

### Interventions

#### The Partners in Parenting Intervention

PiP is a fully automated Web-based parenting program consisting of 3 components [26]. First, parents complete a self-assessment scale (the Parenting to Reduce Adolescent Depression and Anxiety Scale; PRADAS [38]), which assesses their current parenting practices against the Guidelines. Second, based on their responses to the PRADAS, parents receive an individually tailored feedback report outlining their parenting strengths and areas for improvement. The feedback messages are designed to be brief, motivate behavior change, provide practical parenting strategies, and contain links to further information that parents can access if desired. Third, parents are recommended a series of interactive online modules, also based on their responses to the PRADAS. A total of 9 modules are available, and parents can further personalize their program by selecting additional modules (not initially recommended to them) or declining recommended modules. Modules include interactive activities, goal setting exercises, audio clips, vignettes, illustrations, and an end-of-module quiz with immediate feedback, designed to consolidate learning. Each module takes approximately 15 to 25 min to complete. One module is made available to parents every 7 days, to allow sufficient time to complete each module and work on weekly goals before progressing to the next module. Parents were sent automated emails each week to notify them that their next module was available to access via their personalized dashboard. Once parents had completed the initially selected modules, all 9 modules (including those not initially selected) were made available for the remainder of the RCT. Table 1 presents the content covered in the PiP modules and the corresponding sections in the PRADAS, feedback report, and Guidelines. Multimedia Appendix 1 presents screenshots of the intervention.

**Table 1:** Partners in Parenting modules, corresponding sections of the Parenting to Reduce Adolescent Depression and Anxiety Scale (PRADAS) and feedback report and Guidelines subheadings.

Module title and content	Corresponding section of the PRADAS and feedback report	Guidelines subheading^a^
Module: “Connect”—Acknowledges the challenge of connecting with adolescent children and provides specific tips on how to do this.	Your relationship with your teenager	Establish and maintain a good relationship with your teenager
Module: “Nurture roots and inspire wings”—Helps parents establish the important balance between staying involved and interested in their adolescent’s life, while encouraging increasing age-appropriate autonomy.	Your involvement in your teenager’s life	Be involved and support increasing autonomy
Module: “Good friends, supportive relationships”—Provides strategies for parents to support their adolescent’s social skills development.	Your teenager’s relationships with others	Encourage supportive relationships
Module: “Raising good kids into great adults: establishing family rules”—Highlights the importance of consistent and clear boundaries for adolescent behaviors and provides specific strategies to establish these.	Your family rules	Establish family rules and consequences
Module: “Calm versus Conflict”—Addresses the need for adaptive conflict management between parents and between parent and adolescent and provides specific strategies to do these.	Your home environment	Minimize conflict in the home
Module: “Good health habits for good mental health”—Provides strategies to help parents encourage good health habits in their adolescent, including a healthy diet, physical activity, good sleep habits, and abstinence from alcohol and drugs.	Health habits	Encourage good health habits
Module: “Partners in problem solving”—Provides strategies for parents to help their adolescent develop good problem-solving and stress management skills.	Dealing with problems in your teenager’s life	Help your teenager to deal with problems
Module: “From surviving to thriving: helping your teenager deal with anxiety”—Provides strategies for parents to help their adolescent manage their everyday anxiety.	Coping with anxiety	Help your teenager to deal with anxiety
Module: “When things aren’t okay: getting professional help”—Helps parents understand what depression and anxiety problems can look like in adolescents, and what they can do if their adolescent is or becomes unwell.	Getting help when needed	Encourage professional help seeking when needed

^a^Adapted from [34]. Note that 2 of the 11 sections of the Guidelines (*You can reduce your child’s risk of developing depression and clinical anxiety* and *Don’t blame yourself*) do not have specific corresponding sections in the PRADAS or PiP modules, but the key messages they present are included in the feedback report and across all modules.

#### Educational Factsheets (Active Control Condition)

Parents in the control group received access to a series of 5 educational factsheets about adolescent development and mental health. The factsheets provided general information, without individual tailoring or actionable parenting strategies (cf. the PiP intervention). The factsheets were intended to provide information already available to parents as part of a current health promotion approach, with materials adapted from the *Raising Children Network* website [39]. The factsheet topics were as follows: (1) Teen development: an overview, (2) The teenager’s developing brain, (3) The teenager’s changing body, (4) Resilience, and (5) Happy teenagers and teenage wellbeing. The delivery of the factsheets was designed to mirror the delivery of the PiP intervention; parents were emailed once per week with a link to access their next factsheet via their personal dashboard on the trial website, and they had access to the factsheets for the duration of the RCT. The weekly emails occurred at the beginning of the intervention phase (ie, first 5 weeks postbaseline).

### Weekly Check-In Phone Calls

Parents in both groups received a weekly phone call from a member of the research team, commencing 7 days after completion of their baseline assessment. Intervention group parents received 1 phone call per module selected in their program, unless they selected less than 5 modules, in which case, parents received a minimum of 5 calls (to match the control group who received 5 calls). The purpose of the calls was to encourage progress, enhance engagement, provide technical assistance, and answer study-related questions. Research assistants were trained to make the phone calls following a standard script and did not provide individual advice or therapeutic support.

### Measures

#### Primary Outcome Measure 1: The Parenting to Reduce Adolescent Depression and Anxiety Scale

The PRADAS is a self-reported measure of parenting practices across the parenting domains covered in the Guidelines [38] (see Table 1). As a criterion-referenced measure, the PRADAS assesses current parenting practices against specific recommendations in the Guidelines (the *criterion*) and scores parents as either concordant (1) or nonconcordant (0) with the recommendations. The 73 items of the PRADAS cover 8 of the 9 domains of the Guidelines, with one of the original 9 subscales (*relationships with others*) dropped from the scale during the validation process [38]. Most items are scored on a Likert-type frequency (never, rarely, sometimes, and often) or likelihood scale (very unlikely, unlikely, likely, and very likely; for hypothetical scenarios). The item scores are summed to form a total score, ranging from 0 to 73, with higher scores indicating greater concordance with the Guidelines. In a validation study of 711 parents of adolescents aged 12 to 15 years, which included baseline data from the current RCT sample, the total score demonstrated high reliability (agreement coefficient=0.97), acceptable 1-month test-retest reliability (0.78), and convergent validity with 2 existing parenting measures [38]. In the current sample, the agreement coefficient was high at all 3 time points (baseline=0.97, postintervention=0.96, and 12-month follow-up=0.95).

#### Primary Outcome Measure 2: Short Mood and Feelings Questionnaire

The Short Mood and Feelings Questionnaire (SMFQ) is a widely used measure of depressive symptoms in children and adolescents [40]. Both the child (SMFQ-C) and parent (SMFQ-P) versions have 13 items assessing the frequency of depressive symptoms in the past 2 weeks, on a 3-point scale of *not true* (0), *sometimes true* (1), or *true* (2). Item scores are summed to form a total score, ranging from 0 to 26, with higher scores indicative of higher symptom levels. The scale has been documented to have high internal consistency, criterion validity, and convergent validity with other measures of depressive symptoms in children [40-42]. In our sample, both the parent- and child-reported versions had high reliability at all time points, as assessed by coefficient omega (SMFQ-C: baseline omega=0.93, postintervention omega=0.93, 12-month omega=0.95, SMFQ-P baseline omega=0.93, postintervention omega=0.92, 12-month omega=0.94). The correlations between parent and child reports on the SMFQ were *r*=0.48 at baseline, *r*=0.40 at postintervention, and *r*=0.53 at 12-month follow-up (all *P*<.001).

#### Primary Outcome Measure 3: Spence Children’s Anxiety Scale

The Spence Children’s Anxiety Scale (SCAS) is a 39-item child- (SCAS-C) and parent- (SCAS-P) reported measure of child and adolescent anxiety across 6 subscales: separation anxiety, social anxiety, obsessive compulsive symptoms, panic or agoraphobia, generalized anxiety, and fear of physical injury [43,44]. Items are scored on a 4-point scale from 0 (never) to 3 (always) and can be summed to form the 6 subscale scores and a total anxiety score. We calculated the total score, which ranges from 0 to 114, with higher scores representing more anxiety symptoms. The SCAS has been normed on several samples, including Australian school children within the same age range as our sample [45]. Both the parent- and child-reported versions have demonstrated high internal consistency reliability and acceptable test-retest reliability [44-46]. Internal consistency reliability for our sample was high for both versions across the 3 time points (SCAS-C: baseline omega=0.95, postintervention omega=0.96, 12-month omega=0.96, SCAS-P: baseline omega=0.93, postintervention omega=0.94, 12-month ω=0.95). The correlations between SCAS-C and SCAS-P were *r*=0.46 at baseline, *r*=0.43 at postintervention, and *r*=0.47 at 12-month follow-up (all *P*<.001).

#### Secondary Outcome Measure: The Parenting to Reduce Adolescent Depression and Anxiety Scale—Adolescent Report

The Parenting to Reduce Adolescent Depression and Anxiety Scale—Adolescent Report (PRADAS-A) assesses the adolescent’s perspective on the same 8 parenting domains covered in the PRADAS (Cardamone-Breen et al, forthcoming). The scale has fewer items than the PRADAS (total of 43 items), as only items that were developmentally appropriate and could be assessed from the adolescent’s perspective were included. During validation analyses, the *relationships with others* subscale was also removed from the PRADAS-A because of poor psychometric properties (Cardamone-Breen et al, forthcoming). Response options and scoring are similar to the PRADAS, with items assessed on Likert-type scales and scored as either concordant (1) or nonconcordant (0) with the Guidelines’ recommendations. The total score therefore ranges from 0 to 43, with higher scores indicating greater Guidelines-concordant parenting practices. The total score has demonstrated high reliability (agreement coefficient=.97, 3-month test-retest reliability=.81) and moderate correlations with adolescent-reported depression and anxiety symptoms in a validation sample of 670 adolescents aged 12 to 15 years (baseline data from the current RCT was included in the validation study [Cardamone-Breen et al, forthcoming]). The agreement coefficient for the total score in our sample was high (0.97) at all 3 time points. The correlation between the PRADAS and PRADAS-A at each time point was as follows: baseline, *r*=0.26; postintervention, *r*=0.26; and 12-month follow-up, *r*=0.33 (all *P*<.01).

#### Intervention Adherence, Completion, and Access During Follow-Up Period

Intervention adherence was operationalized as the percentage of parents who completed their program as intended, calculated as 100% × [(number of parents whose observed usage equals their intended usage)/(number of parents who received the intervention)] [34,47]. Intervention completion was defined as the percentage of the intended program that was completed, that is, intervention completion=[100% × (observed usage)/(intended usage)]. For the intervention group, observed usage was defined as the number of modules completed, and intended usage as the number of modules initially selected. For the control group, intended usage was defined as reading all 5 factsheets, and observed usage was defined as the number of factsheets that had been opened by the parent (determined by timestamps stored in the system when parents clicked the link to open their factsheet). We also examined whether parents completed their program during the active intervention phase, which was defined as the time between the parent baseline assessment and the adolescent postintervention. If the adolescent did not complete the assessment, the date of the parent postintervention assessment was used. Finally, we examined the number of parents who accessed their program after the active intervention phase (ie, between postintervention and 12-month assessment time points).

### Procedures

#### Registration and Baseline Assessments

Parent participants registered themselves and their adolescent via the dedicated trial website and provided consent and contact details for their adolescent (see Multimedia Appendix 2 for informed consent documentation). Email verification was required at this point. A member of the research team then phoned the adolescent to inform them of the study requirements and obtain assent (if they agreed to participate). Parents were not informed of their adolescent’s decision to participate and could continue in the study regardless of adolescent participation. If the adolescent declined to take part, the adolescent assessments were cancelled so that parent participation could proceed as per protocol. Adolescents who agreed to participate were guided through completion of their online baseline assessment over the phone, with the researcher providing assistance as required. On completion of the adolescent baseline assessment (or cancellation of the assessment by a researcher), the trial website automatically generated an email invitation to the parent, inviting them to complete their baseline assessment. Parents were then required to log on to the website in their own time to complete their baseline assessment. Parent and adolescent assessments included their respective versions of the PRADAS, SCAS, and SMFQ.

#### Randomization and Blinding

Immediately following completion of the parent baseline assessment, parents were automatically allocated to the intervention or control condition using a computer-generated unblocked, unstratified randomization procedure, with a 1:1 allocation ratio. At this point, the intervention group parents were presented with their individually tailored feedback onscreen and were emailed a PDF copy of the feedback report. Control group parents were presented with their first factsheet. Therefore, parents were not blinded to their allocation nor were the researchers who spoke to parents during weekly check-in phone calls. Adolescents were not informed of their parent’s allocation and, therefore, were assumed to be blinded. As all assessments were completed online via the dedicated trial website, blinding of assessor was not relevant.

#### Follow-Up Assessments

The procedure for 3- and 12-month follow-up assessments was similar to the baseline procedure. Both parents and adolescents were reimbursed with an Aus $15 electronic voucher for completion of each of the 3- and 12-month follow-up assessments.

#### Adolescent Symptom Elevation Procedure

At all time points, the participants were followed up by a member of the research team if both the parent and adolescent reported elevated symptoms on the SCAS or SMFQ, based on predetermined cutoff scores. For the SCAS, this was defined as a total score greater than or equal to 1.5 SDs above the mean based on Australian community sample norms [48]. For the SMFQ, scores greater than or equal to 8 were considered elevated [40]. Follow-up actions included email notifications to parents alerting them to their adolescent’s elevated symptoms and providing avenues for seeking professional support (n=38 at baseline, n=25 at postintervention, and n=28 at 12-month follow-up; no significant differences between groups). Adolescents who reported particularly high scores on the SMFQ (SMFQ-C total score >20) were also phoned by a postgraduate clinical psychology student, who conducted a risk assessment and provided referral information as required (n=8 at baseline, n=5 at postintervention, and n=10 at 12-month follow-up; no significant differences between groups).

### Statistical Methods

Less than 4% of participants had missing data on any measures. Item level missing data were replaced with the participant’s mean response on the corresponding subscale for cases with less than 23% missing data on a given measure. This is considered an appropriate method of imputation for this amount of missing data [49]. For cases with greater than 23% of missing items on a measure, the measure was considered missing entirely and excluded from analyses.

Analyses were conducted in SPSS version 25 (IBM Corp), with an a priori alpha level of .05. To assess for potential attrition biases, we compared participant characteristics and scores on baseline and postintervention outcome measures between those who completed 12-month follow-up assessments and those who did not. Independent samples *t* tests (for continuous variables) and chi-square analyses (for categorical variables) were used for these analyses. We also examined potential group differences in follow-up actions taken for adolescents who reported elevated symptoms at postintervention.

Primary and secondary outcome analyses were conducted on an intention-to-treat basis, using mixed-model repeated measures (MMRM), with an unstructured covariance matrix. MMRM uses all the available data from all the participants, including those who withdrew from follow-up assessments [50]. It is a preferred analytic approach for repeated-measures designs when data are considered missing at random or missing completely at random [50,51]. As our hypotheses related to change from baseline to 12-month follow-up, we specified planned contrast tests of the group × measurement-occasion interaction from baseline to 12-month follow-up, within the overall group × measurement-occasion mixed model. This was the primary result of interest. Pairwise comparisons between groups at trial endpoint (12-month follow-up) were also examined. Cohen *d* effect sizes with 95% CIs are reported for all analyses.

Owing to the positive skew of model residuals for the SCAS and SMFQ at all occasions, a square-root transformation was applied, which improved the distribution of residuals. All analyses were repeated using the transformed data to check robustness of the results. For most analyses, the results did not change with transformation. When the results did differ, the overall conclusions were made by considering the findings based on both raw and transformed data. For ease of interpretation, raw data have been plotted, with footnotes to indicate where results differed with transformation.

To assess for potential mediation of intervention effects on adolescent symptoms by change in parenting, we conducted simple mediation analyses using the PROCESS macro for SPSS [52,53]. Separate mediation models were run for each symptom measure (ie, outcome variable), with 5000 bootstrap samples for bias-corrected bootstrap 95% CIs. In each model, group (coded as 0= control, 1=intervention) was entered as the predictor variable, 12-month symptom measure score as the outcome variable, and postintervention PRADAS score as the mediator variable. Baseline PRADAS and baseline symptom measure score (corresponding to the outcome variable) were entered as covariates.

Finally, we conducted post hoc moderation analyses to explore moderation of intervention effects of adolescent age, gender, and baseline symptoms on outcomes. For age and baseline symptoms, the continuous moderator variable (ie, child age at registration or baseline symptom score) was added to the mixed model as a covariate, including a 3-way interaction term (ie, group × measurement-occasion × moderator) whose significance constituted a test of a differential effect of the moderator on outcome of the intervention. Significant moderation effects were interpreted using estimated marginal means plotted for values of the covariate (ie, moderator variable) at its 25th and 75th percentile at baseline in the sample. To minimize shared method variance effects for moderation analyses using baseline symptom measures, we used the symptom measure reported by the opposite informant as the moderator variable (eg, for the outcome of SMFQ-P, baseline SMFQ-C score was used as the moderator). For the parenting measures, we conducted 2 moderation analyses, with each of the symptom measures (SCAS and SMFQ, opposite informant to outcome measure) entered in separate models. Moderation by child gender was assessed in a similar manner using gender as an additional factor rather than as a covariate.

## Results

### Sample Characteristics

The sample’s characteristics have been reported in detail elsewhere [34] and included in Multimedia Appendix 3. Parent participants were predominantly female (87.2%), were married or in a de facto relationship (76.6%), were employed full or part time (86.6%), spoke English as their primary language at home (84.1%), were from an intact family situation (70.5%), and had tertiary-level education (58.2%). Parents had a mean age of 45.15 years (SD 5.20) and their adolescents (55.4% male) had a mean age of 13.68 years (SD 1.06). In addition, 59.1% of the parents reported having a current or past history of mental illness, whereas less than a quarter of adolescents were reported by their parents to have a current (18.9%) or past (15.9%) mental health diagnosis.

### Attrition

**Figure 1:**
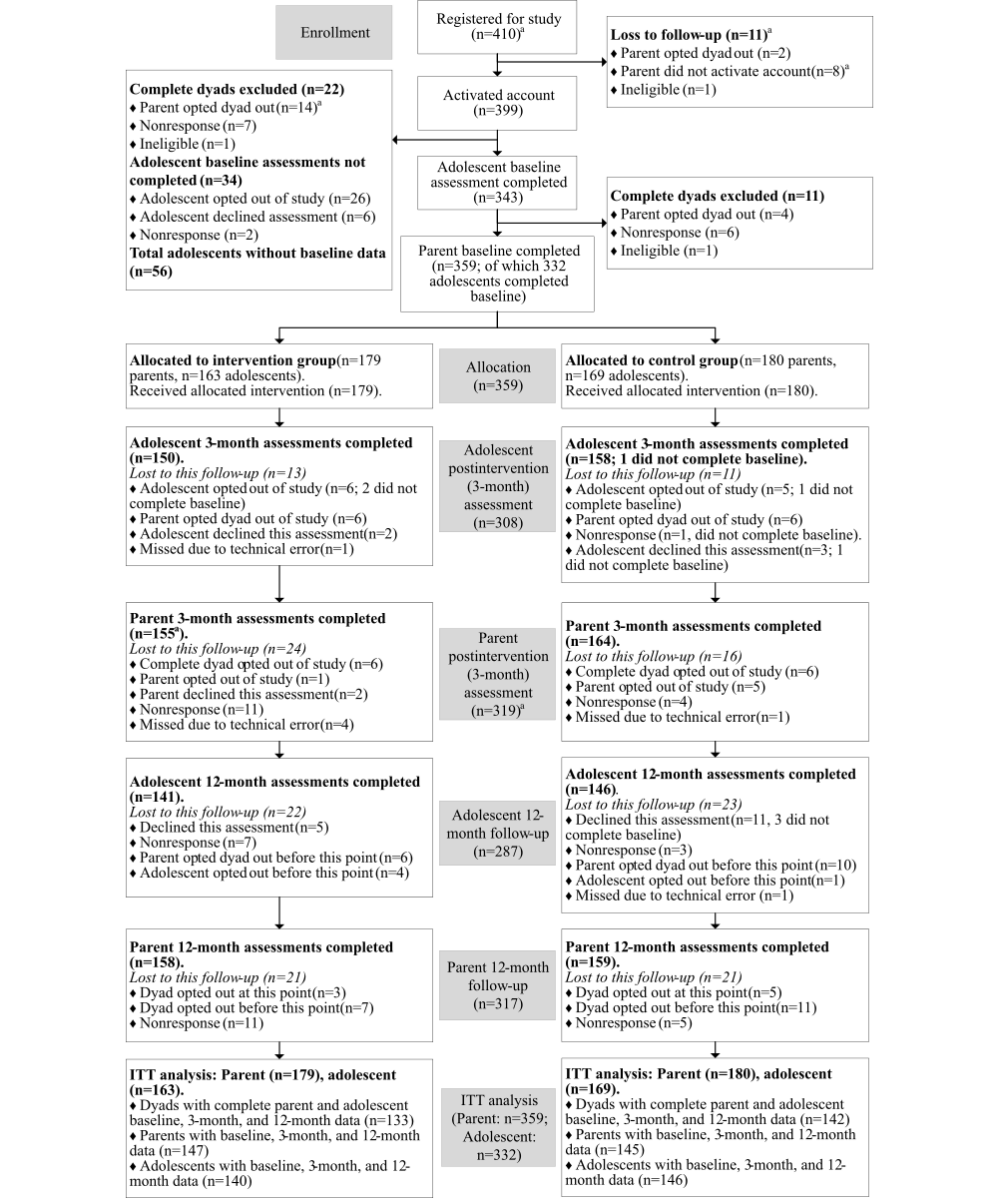
Consolidated Standards of Reporting Trials (CONSORT) participant flow diagram. (a) These numbers differ from those published in the postintervention paper because of errors detected when preparing the 12-month follow-up data.

As shown in Figure 1, of the 359 parent participants who completed the baseline assessments and were randomized, 319 completed postintervention assessments and 317 went on to complete 12-month follow-up (intervention group n=158, control group n=159). The number of parents reported to complete postintervention differs from the original RCT outcome paper (previously reported as n=318) because of an error detected when preparing the 12-month data. A parent in the intervention group was excluded from the original paper because of missing individual item response data (missing 9 items [12.3%] of the postintervention PRADAS); however, in this paper, the missing items were imputed, allowing this participant to be included in the analyses. For adolescent participants, 332 completed baseline assessments, 308 completed postintervention assessments, and 287 completed 12-month follow-up. Therefore, the attrition rate at 12-month follow-up was 11.7% for parents and 13.6% for adolescents. This did not differ between conditions for parents (11.7% in each group) or adolescents (intervention group: 13.5%; control group: 13.6%). Figure 1 presents the participant flow diagram.

We examined demographic characteristics and scores on baseline and postintervention measures between participants who completed 12-month follow-up and those who did not. There were no differences on any baseline measures between completers and noncompleters nor were there differences in parent or child demographic characteristics, with the exception of parent education level. Parents who completed the 12-month assessment were more likely to have tertiary-level education than those who did not (χ^2^_1_, [N=359]=9.9; *P*=.002). In addition, adolescents of parents who completed the 12-month follow-up had significantly higher PRADAS-A scores (mean 23.94, SD 6.20) at postintervention compared with adolescents of parents who did not complete 12-month follow-up (mean 21.05, SD 6.84; *t*_(306)_=2.05; *P*=.04).

### Time Interval Between Assessments

The mean time interval between parent baseline and 12-month follow-up was 385.71 days (SD 19.16, median 380.04, range 361-487 days), and the mean interval between parent postintervention and 12-month assessments was 266.66 days (SD 35.43, median 273.92, range 86-366 days). For adolescent assessments, the mean interval between baseline and 12-month follow-up was 376.59 days (SD 13.19, median 371.98, range 362-452) and between postintervention and 12-month follow-up was 266.41 days (SD 27.91, median 273.97, range 87-315). As reported in the postintervention paper [34], the wide range in time intervals was because of a technical error resulting in delayed postintervention assessments for 59 dyads. The time intervals did not differ significantly between groups (all *P*>.05). There was an average interval of 12.14 days between completion of the adolescent 12-month assessment and the parent 12-month assessment (SD 13.18, median 7.16, range 0-57). At 12-month follow-up, dyads in the control group had a significantly shorter interval between completion of adolescent and parent assessments (control group: mean 9.94 days, SD 12.22; intervention group: mean 14.47 days, SD 13.80; *t*_(265.54)_=−2.88; *P*=.004).

### Intervention Completion and Adherence

As reported previously, the mean intended program use by the intervention group (n=179) was 6.85 out of a possible 9 modules. The mean observed usage by the intervention group was 5.17 modules [34]. At the time of data extraction for this paper, parents in the intervention group had completed an average of 73.7% of their selected program. Intervention adherence in the intervention group was 44.1% (n=79 parents whose observed usage equaled their intended usage). During the follow-up period (from 3-12 months postbaseline), 12 of the 179 intervention-group parents (6.7%) accessed a mean of 2 modules (range 1-8, SD 2). In the control group, 33 of the 180 parents (18.3%) accessed a mean of 2 factsheets (range 1-4, SD 1.20).

### Primary Outcome Measure 1: Parenting to Reduce Adolescent Depression and Anxiety Scale

**Figure 2:**
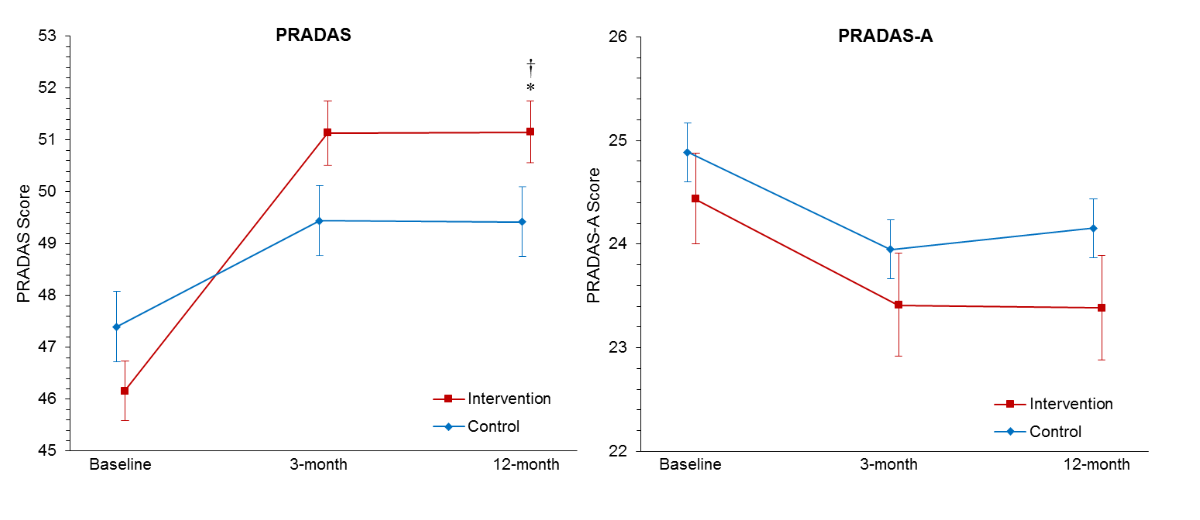
Estimated marginal means for Parenting to Reduce Adolescent Depression and Anxiety Scale (PRADAS) and PRADAS—Adolescent (PRADAS-A) Report scores at baseline, postintervention (3-months postbaseline), and 12-month follow-up, estimated under the group × measurement-occasion mixed model. Error bars represent SEs. Higher scores on the PRADAS and PRADAS—Adolescent Report indicate greater concordance with the parenting guidelines (ie, more protective parenting factors and fewer parenting risk factors). Planned contrast of interaction (baseline to 12 months) was significant, *P*<.001. Pairwise comparison of group difference at 12-month follow-up was significant, *P*=.04.

Observed scores for all outcome measures at each measurement occasion are presented in Multimedia Appendix 4. Table 2 displays the planned contrast results of the group × measurement-occasion interaction from baseline to 12-month follow-up for all primary and secondary outcome analyses. As shown in Table 2, there was a significant group-by-time interaction for the PRADAS, *t*_328.17_=4.81, *P*<.001, with the intervention group showing a significantly greater increase in PRADAS scores from baseline to 12-month follow-up compared with controls (see Figure 2). The effect size of the interaction was medium (Cohen *d*=0.51; 95% CI 0.30 to 0.72). Pairwise comparisons of the 2 groups at 12-month follow-up also indicated a significant group difference (*F*_1,347.77_=4.11; *P*=.04), although the effect size was small (Cohen *d*=0.21; 95% CI −0.01 to 0.43).

#### Primary Outcome Measures 2 and 3: Adolescent Anxiety and Depression Symptoms

**Figure 3:**
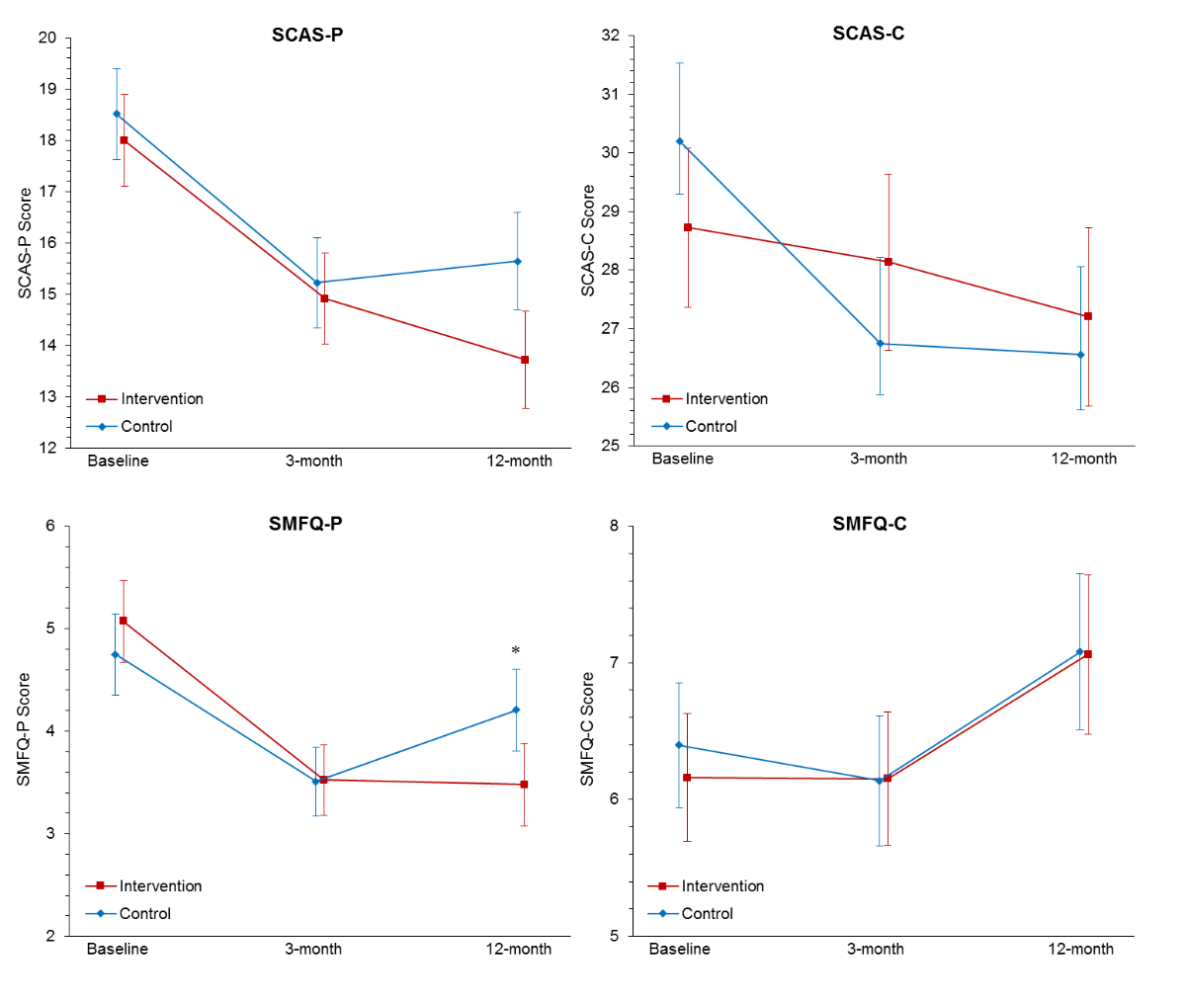
Estimated marginal means for the SCAS—Parent Report (SCAS-P), SCAS—Child Report (SCAS-C), SMFQ—Parent Report (SMFQ-P), and SMFQ—Child Report (SMFQ-C) estimated under the group × measurement-occasion mixed model. Error bars represent SE. *Planned contrast of baseline to 12-month group × measurement-occasion interaction effect significant at *P*<.05 level, when square-root-transformed data were used. SCAS: Spence Children’s Anxiety Scale; SMFQ: Short Mood and Feelings Questionnaire.

As shown in Table 2 and Figure 3, there was a significant group x measurement occasion interaction from baseline to 12-month follow-up for the SMFQ-P, when transformed data were used (Cohen *d*=−0.21; see Multimedia Appendix 5 for MMRM results on transformed data). However, when analyses were run on raw data, there was no significant interaction for any of the symptom measures (all *P*>.05). Using transformed data, there was a significant main effect for time over the 3 measurement occasions for the SCAS-P, SCAS-C, and SMFQ-P (all *P*<.001) but not for the SMFQ-C (*P*=.12). When raw data were used, there was a significant main effect for time for all symptom measures (SCAS-P and SMFQ-P, *P*<.001; SCAS-C, *P*=.001; SMFQ-C, *P*=.009; see Figure 3). For the parent-reported measures, both groups showed a significant reduction from baseline to postintervention, after which the groups appeared to diverge from postintervention to 12-month follow-up (see Figure 3). On the SMFQ-P, the increase from postintervention to 12-month follow-up was significant for the control group (only on raw data), whereas the intervention group remained stable (no significant difference between 3 and 12 months). The reduction from baseline to 12-month follow-up was significant for both groups on the SCAS-P, as well as for the intervention group on the SMFQ-P and the control group on the SCAS-C. When transformed, the reduction from baseline to 12-month follow-up was also significant for the control group on the SMFQ-P and the intervention group on the SCAS-C. On the SCAS-C, the control group demonstrated a significant reduction from baseline to postintervention (with both raw and transformed data). Pairwise comparisons indicated no significant group differences on any of the symptom measures at any time point, with both raw and transformed data (all *P*>.05).

**Table 2:** Mixed-model repeated measures planned contrast test of group × measurement-occasion interaction from baseline to 12-month follow-up for all primary and secondary outcome measures.

Outcome measure		Estimated marginal means (SE)		t test *(df)*^a^	*P* value	*d*_interaction_ (95% CI)^b^	*d*_12 months_ (95% CI)^c^
		Intervention	Control				
**Parenting to Reduce Adolescent Depression and Anxiety Scale**							
	Baseline	46.58 (0.57)	47.88 (0.57)	—^d^	—	—	—
	12 months	51.68 (0.59)	49.99 (0.61)	4.81 (328.17)	<.001	0.51 (0.29 to 0.71)	0.21 (−0.01 to 0.43)
**Parenting to Reduce Adolescent Depression and Anxiety Scale—Adolescent Report**							
	Baseline	24.44 (0.44)	24.89 (0.43)	—	—	—	—
	12 months	23.38 (0.51)	24.16 (0.50)	−0.59 (297.53)	.56	−0.06 (−0.28 to 0.15)	−0.13 (−0.36 to 0.11)
**Spence Children’s Anxiety Scale—Parent Report**							
	Baseline	17.99 (0.90)	18.51 (0.90)	—	—	—	—
	12 months	13.72 (0.95)	15.64 (0.94)	−1.28 (321.37)	.20	−0.14 (−0.37 to 0.08)	−0.12 (−0.34 to 0.10)
**Spence Children’s Anxiety Scale—Child Report**							
	Baseline	28.73 (1.36)	30.20 (1.33)	—	—	—	—
	12 months	27.20 (1.52)	26.56 (1.49)	1.36 (294.14)	.18	0.16 (−0.07 to 0.39)	0.04 (−0.19 to 0.27)
**Short Mood and Feelings Questionnaire—Parent Report**							
	Baseline	5.07 (0.40)	4.75 (0.40)	—	—	—	—
	12 months	3.48 (0.40)	4.21 (0.40)	−1.88 (329.02)	.06^e^	−0.21 (−0.42 to 0.01)	−0.14 (−0.36 to 0.08)
**Short Mood and Feelings Questionnaire—Child Report**							
	Baseline	6.16 (0.47)	6.40 (0.46)	—	—	—	—
	12 months	7.06 (0.58)	7.08 (0.57)	0.33 (296.86)	0.74	0.04 (−0.19 to 0.27)	−0.01 (−0.24 to 0.22)

^a^*t* statistic of the planned contrast test of group × measurement-occasion interaction from baseline to 12-month follow-up, estimated under the group × measurement-occasion mixed model.

^b^Cohen *d* effect size of the group × measurement-occasion interaction from baseline to 12-month follow-up, calculated based on the *t* statistic of the planned contrast. Negative effect size indicates greater reduction in scores from baseline to 12-month follow-up in the intervention group compared with the control group.

^c^Cohen *d* effect size of the difference between groups at 12-month follow-up. Negative effect size indicates lower scores in the intervention group compared with the control group.

^d^Not applicable.

^e^Becomes statistically significant with square-root-transformed data: *t*_(324.98)_=−2.04; *P*=.04; *d*=−0.21 (95% CI −0.42 to −0.01).

#### Secondary Outcome Measure: Parenting to Reduce Adolescent Depression and Anxiety Scale—Adolescent Report

The planned contrast of the group × measurement-occasion interaction from baseline to 12-month follow-up was not significant for the PRADAS-A (see Table 2 and Figure 2). There was a significant main effect for time, with both groups reporting significantly reduced PRADAS-A scores over the 3 occasions: *F*_(2,297.99)_=9.27; *P*<.001. However, the comparisons of group differences at each occasion were not significant (all *P*>.05).

### Mediation Analyses

Mediation analyses revealed that the indirect effect of group on 12-month SMFQ-P via postintervention PRADAS was significant (indirect effect *b*=−0.08; 95% CI −0.16 to −0.01) when transformed symptom data were used. However, no significant mediation was found for any of the 4 symptom measures when using raw data (all *P*>.05; see Multimedia Appendix 6 and Table 1).

### Post Hoc Moderation Analyses

#### Parental Concordance With the Guidelines (Parenting to Reduce Adolescent Depression and Anxiety Scale and Parenting to Reduce Adolescent Depression and Anxiety Scale—Adolescent Report), Moderated by Baseline Symptom Levels

Results of the PRADAS moderation analyses suggested that neither child anxiety (SCAS-C) nor depression (SMFQ-C) symptoms at baseline moderated intervention effects on parent reports of parenting (*P*>.05; see Multimedia Appendix 7). For adolescent reports of parenting (PRADAS-A), results suggested that baseline parent-reported anxiety (SCAS-P) moderated intervention effects on PRADAS-A: *F*_(2, 296.81)_=10.23, *P*<.001. Figure 1 in Multimedia Appendix 7 presents the estimated marginal means of PRADAS-A, with baseline SCAS-P calculated at the 25th and 75th percentiles. As shown in this figure, among adolescents whose parents reported higher baseline SCAS-P scores, adolescents whose parents received PiP reported a greater reduction in PRADAS-A scores over time than controls. The analysis of PRADAS-A moderated by baseline SMFQ-P was not significant (*P*>.05). Multimedia Appendix 7 presents the results of these analyses.

#### Adolescent Depression and Anxiety Symptoms Moderated by Baseline Symptom Levels

We conducted separate MMRMs for each of the symptom measures, moderated by baseline scores on the opposite informant of the same measure. Of the 4 analyses, only the model of SMFQ-P moderated by baseline SMFQ-C was significant: *F*_(2, 295.27)_=4.40, *P*=.01. However, as shown in Multimedia Appendix 7 (Figure 2), this moderation effect may be attributable to baseline differences.

#### Adolescent Age and Gender as Moderators

We ran separate MMRMs for all 6 outcome measures, moderated by adolescent age at baseline and gender (in separate models). None of the 3-way interactions were significant (all *P*>.05; results available from the first author upon request).

## Discussion

### Principal Findings

#### Parent Reports of Parenting

This RCT evaluated the medium-term effects of PiP. Consistent with our hypothesis, when compared with parents who received an educational-factsheet control intervention, parents who received PiP reported greater improvements in their parenting behaviors from baseline to 12 months later. These improvements represent reductions in parental risk factors and increases in parental protective factors for adolescent depression and anxiety [26,38]. Notably, the effect was medium in size, similar to that found at postintervention [34]. This suggests that the effect of PiP on parenting factors was maintained up to 9 months after the intervention and reduces the likelihood of social desirability and learning or priming effects (eg, receiving PiP alerts parents to *good parenting*, which influences their responses to the PRADAS at postintervention). The observed medium-term effect was despite minimal access to PiP modules by parents between the postintervention and 12-month assessments, so the maintenance of effects is unlikely because of *booster* effects.

#### Adolescent Symptoms

Contrary to expectations, at 12-month follow-up, there was no robust indication that PiP significantly reduced adolescent depression and anxiety symptoms as reported by either parents or adolescents, compared with the active-control intervention. This is largely consistent with findings at postintervention [34], suggesting that the effect of PiP on parent-reported parenting did not translate into significant reductions in adolescent symptoms compared with an active control. The effect of PiP on parent-reported depressive symptoms was significant (with a small effect size) when the analysis was conducted using transformed data, but it only approached significance when based on raw data. This suggests that PiP may lead to greater reduction in adolescent depressive symptoms than the control intervention, but given that this parent-reported finding was not verified by adolescent reports or other objective measures, the efficacy of PiP on adolescent depressive symptoms remains inconclusive. Nonetheless, there was a similar pattern of change over time for both parent-reported depression symptoms and anxiety symptoms, whereby the intervention and control groups appeared to diverge after postintervention, with symptoms among the intervention group remaining stable, whereas symptoms in the control group started to increase over time. The pattern of change in the control group reflects an increase in depressive symptoms that is commonly seen in epidemiological research as adolescents approach mid-to-late adolescence, when the onset of depression and some anxiety disorders peaks [1]. A longer-term follow-up is required to ascertain whether these findings persist and demonstrate a long-term preventive effect of PiP.

It is possible that the use of an active control in this study, although a methodological strength, may have obscured the benefits of PiP on adolescent internalizing outcomes. Although the educational factsheets in the control intervention did not provide specific personalized parenting strategies in an engaging format (like PiP), they did provide credible information on adolescent development and general advice for parents about how they can best support their adolescent’s development and well-being. For a highly educated and motivated sample of parents, this may be sufficient to produce the improvement in parenting observed in the control group, albeit to a weaker extent than the intervention group, which was maintained at 12-month follow-up. In turn, the improvement in parenting in both groups may explain the significant reduction in reported symptoms (except adolescent-reported depressive symptoms) over time in both groups and, hence, the nonsignificant group difference in change over time. This is notable because many preventive interventions for adolescent internalizing disorders that have demonstrated significant effects were compared with nonactive or no-intervention control groups [18,54]. Although this study’s findings do not support the hypothesized superiority of PiP over an active control condition, it is notable that such brief, self-guided online parenting interventions were able to produce significant reductions in adolescent depressive and anxiety symptoms over time. These findings are promising given the scalability of PiP and the argument that even small reductions in mental health symptoms at the individual level could translate to significant population health benefits [55].

#### Adolescent Reports of Parenting

Contrary to expectation, a significant improvement in parenting was not seen in adolescent reports. This is consistent with findings at postintervention [34], suggesting that the effect of PiP on parent-reported parenting did not translate into adolescent perceptions of parenting. Though the discrepancy in findings involving parent-reported versus adolescent-reported parenting is notable, it concurs with most previous research involving parent and adolescent informants of parenting [56]. The PiP intervention components were tailored based on parents’ responses to the PRADAS, without taking into account the adolescents’ perspective (ie, their responses on the PRADAS-A). In particular, to personalize the feedback report for each parent and to identify the modules to recommend to the parent, PiP uses the parent’s responses on the PRADAS to identify their strengths and areas from improvement. As such, insofar as the parent and adolescent perceptions of parenting differ, PiP may have targeted some parenting behaviors not perceived by adolescents to need improvement and failed to target other parenting behaviors that from the adolescent’s perspective, need improvement. Post hoc analyses of 334 parent-adolescent dyads in this sample who completed the PRADAS and PRADAS-A support this possibility. On the basis of parent reports only, a mean of 6.71 modules (SD 1.53, range 1-9) were recommended, and a mean of 26.63 feedback messages (SD 7.81, range 7–60) were provided to parents. If the adolescents’ perspectives were also taken into account, the mean number of modules recommended would have increased to 7.94 (SD 1.06, range 4-9), and a mean of 35.27 (SD 7.67, range 17-65) feedback messages would have been provided. Future research is required to examine whether tailoring PiP to both parent and adolescent perspectives of parenting can enhance the effects of PiP, especially on adolescent-reported parenting and symptom outcomes.

Divergence in parent and adolescent reports of parenting, especially during early adolescence, is well established and considered to be normative in the developmental literature [56]. From a developmental perspective, maturational processes during adolescence, such as autonomy seeking and individuation, may mean that parents and adolescents experience their interactions differently [57]. Nonetheless, it remains possible that parents’ perceived improvements in their parenting were not observed by their adolescents up to 9 months after the intervention because parent-reported parenting changes did not translate into tangible behavioral changes observed by the adolescent. More recently, researchers have underscored the value of examining the degree of parent-adolescent discrepancy as a window into the dynamics of the parent-adolescent relationship and its associations with adolescent development and adjustment [58]. Further research is required to examine whether parent-adolescent discrepancies in perceptions of parenting may account for or moderate the effects of the PiP intervention on adolescent symptom outcomes.

### Mediation and Moderation Analyses

Results from the mediation analyses were generally consistent with the above findings. Only the mediation hypothesis for parent-reported adolescent depressive symptoms was supported when using transformed data, whereby PiP compared with the control intervention led to greater improvements in parent-reported parenting from baseline to postintervention, which led to greater reductions in parent-reported adolescent depressive symptoms from baseline to 12-month follow-up. Consistent with the parenting pathway proposed by Sandler et al (2011), this mediation suggests that PiP’s effects on parenting may account for a longer-term benefit on adolescent depressive symptoms. Future research is required to examine whether this preliminary finding would be corroborated by other measures of adolescent functioning, such as school engagement and academic performance.

In our post hoc moderation analyses, the moderation effect of adolescent-reported baseline depressive symptoms found at postintervention [34] emerged again, suggesting that among adolescents with elevated depressive symptoms at baseline, PiP was more effective than the control in reducing parent-reported depressive symptoms. However, upon probing, it appears that the interaction effect may be largely accounted for by baseline differences between intervention and control groups. Hence, it remains to be ascertained whether the moderation effect supports the utility of PiP as an indicated prevention program. Adolescent age and gender did not significantly moderate intervention effects, suggesting that PiP’s effects may be similar across early-to-mid-adolescence and for male and female adolescents.

### Comparison With Previous Work

On the basis of the findings of a recent systematic review of technology-assisted preventive parenting interventions [27], PiP is the only online intervention aimed at preventing adolescent internalizing disorders. According to Yap et al’s review [18], the only other universal preventive intervention for parents of adolescents is Tuning in to Teens (TINT), a group parenting program targeting emotion socialization [19]. In an RCT comparing TINT with a no-intervention control [19], TINT was found, at about 9 months postintervention, to produce a moderately large effect on parent-reported parenting, a small-to-medium effect on adolescent-reported parenting, small-to-medium effects on anxiety symptoms (small effect based on adolescent reports, small-to-medium based on parent reports), and a small-to-medium effect on parent-reported depressive symptoms (nonsignificant effect based on adolescent reports). A few observations are notable when comparing TINT with PiP, taking into consideration the differences in the comparison group (no-intervention versus active control, respectively) and modality of the intervention (face-to-face vs Web-based). First, the effects of PiP on parent-reported parenting compare favorably with those of TINT. Second, the Web-based self-guided modality of PiP may not provide an adequate intervention to produce changes in parenting behavior that are noticeable by adolescents, to in turn produce robust reductions in their internalizing outcomes. In contrast, the higher-intensity modality of TINT provides opportunities for parents to practice and discuss learned parenting behaviors with the facilitators and other parents, which may consolidate their learning into tangible changes in their behaviors when interacting with their adolescents. Further research is required to investigate whether a more-intensive, guided version of PiP (eg, providing additional coaching support via phone or videoconferencing; [26]) can yield stronger and more robust benefits on adolescent internalizing outcomes and adolescent-reported parenting.

The findings of PiP’s effects to date share some similarities to those of other preventive parenting interventions that found developmental cascading effects at long-term follow-up (at least 5-6 years postintervention [35,37]). Specifically, intervention effects on parenting were found at postintervention and maintained into the 12-month follow-up; effects on adolescent depressive symptoms were observed at postintervention only among adolescents with elevated symptoms at baseline, but by the 12-month follow-up, the effect of PiP on depressive symptoms appeared to be emerging across the randomized sample, albeit not robustly. The earlier follow-up assessments for the New Beginnings [35] and Family Check-Up [37] RCTs had similarly promising but inconclusive findings, yet their subsequent follow-up assessments then found significant long-term benefits of the programs across various functioning domains. As such, a longer-term follow-up of this RCT is warranted to test whether PiP has developmental cascading effects on adolescent outcomes in late adolescence and early adulthood.

### Strengths and Limitations

This study had various strengths. It evaluated a world-first tailored Web-based parenting intervention to prevent adolescent internalizing problems, using a rigorously designed RCT with an active control group, parent and adolescent informants on all outcomes of interest, low attrition rates that are balanced across groups, and high intervention completion. However, various limitations merit comment. First, despite successfully recruiting a large community sample, there was overrepresentation by mothers and highly educated parents. Although this is a limitation shared by most online preventive parenting interventions [27], it urgently needs to be redressed so that the dissemination of evidence-based online interventions does not perpetuate the exclusion of fathers and of parents from vulnerable or disadvantaged backgrounds, inadvertently contributing to the widening of social inequalities in health between families of higher versus lower socioeconomic positions [59]. Second, for reasons of parsimony, this study only included 1 parent and 1 adolescent per family. Nonetheless, we encouraged parent participants who had a co-parenting partner to share and discuss the resources they received to enhance consistency in co-parenting. It remains to be seen whether providing both parents with the intervention will yield synergistic benefits, as suggested by previous research [60]. Third, parents were allowed to participate in the trial if their adolescent declined participation, resulting in a small subset of our sample (37/359, 10.3%) without data on self-reported adolescent symptom outcomes. We chose to have inclusive eligibility criteria to capture a more representative and diverse sample, given the pragmatic design of the trial. Thus, the small loss of data is outweighed by the greater generalizability of our findings to a real-world implementation setting, where adolescents would not be required to participate with their parent. Fourth, this study did not include behavioral measures of parenting or other measures of adolescent functioning outcomes, including quality of life, school engagement, and academic achievements, as well as measures of cost-effectiveness. Future research using these measures will provide insights into whether PiP produces change in objectively measured parenting behaviors and yields broader benefits for adolescents beyond the reduction of internalizing symptoms. Evidence of its cost-effectiveness is also important for advancing the prevention agenda, given that prevention research and interventions are still largely underfunded even in developed countries, including Australia [61]. Fifth, we assumed that adolescents were unaware of their parent’s group allocation, but we did not implement checks to verify the blinding nor did we ask parents to conceal their program from their adolescent. Finally, given that the onset of depression and some anxiety disorders peaks in mid-to-late adolescence, a longer-term follow-up examining cases of disorder is required to adequately test whether PiP can prevent the onset of depression and anxiety disorders across adolescence and into early adulthood. Such evidence is notably lacking in preventive parenting intervention research [18,27].

### Conclusions

Overall, this study found that the PiP intervention produced significantly greater improvements than an active control in parenting risk and protective factors associated with adolescent risk for depression and anxiety. The effects persisted for up to 9 months postintervention, reducing the likelihood that they were due to social desirability or short-term priming effects. Findings from the analyses using transformed data and from mediation analyses suggest that PiP may have some benefits for adolescent symptoms that need to be ascertained in future research. However, parent-reported changes in parenting were not reported by adolescents, and there were no robust findings with regard to reductions in adolescent symptoms. Nonetheless, significant reductions in adolescent symptoms were observed over time in both groups. Given these promising findings, the paucity of evidence-based resources for parents of adolescents, and the scalability of the Web-based platform, PiP may be useful as a low-cost, sustainable public health universal prevention program to empower parents for their adolescents’ mental health.

## Appendix

Multimedia Appendix 1 Screenshots of the Partners in Parenting intervention.

Multimedia Appendix 2 Participant informed consent documentation.

Multimedia Appendix 3 Sample characteristics at baseline.

Multimedia Appendix 4 Observed scores for all measures at each measurement occasion.

Multimedia Appendix 5 Results of primary and secondary outcome mixed-model repeated measures, run on square-root-transformed data.

Multimedia Appendix 6 Results of mediation analyses.

Multimedia Appendix 7 Results of posthoc moderation analyses.

Multimedia Appendix 8 Consolidated Standards of Reporting Trials (CONSORT) E-Health (V 1.6.1) checklist.

## Abbreviations

MMRM: mixed-model repeated measures
NHMRC: National Health and Medical Research Council
PiP: Partners in Parenting
PRADAS: Parenting to Reduce Adolescent Depression and Anxiety Scale
PRADAS-A: Parenting to Reduce Adolescent Depression and Anxiety Scale—Adolescent Report
RCT: randomized controlled trial
SCAS: Spence Children’s Anxiety Scale
SCAS-P: Spence Children’s Anxiety Scale—Parent Report
SCAS-C: Spence Children’s Anxiety Scale—Child Report
SMFQ: Short Mood and Feelings Questionnaire
SMFQ-P: Short Mood and Feelings Questionnaire—Parent Report
SMFQ-C: Short Mood and Feelings Questionnaire—Child Report
TINT: Tuning in to Teens

## References

[1] Kessler RC, Petukhova M, Sampson NA, Zaslavsky AM, Wittchen HU (2012). Twelve-month and lifetime prevalence and lifetime morbid risk of anxiety and mood disorders in the United States. Int J Methods Psychiatr Res.

[2] Woodward LJ, Fergusson DM (2001). Life course outcomes of young people with anxiety disorders in adolescence. J Am Acad Child Adolesc Psychiatry.

[3] Rao U, Ryan ND, Birmaher B, Dahl RE, Williamson DE, Kaufman J, Rao R, Nelson B (1995). Unipolar depression in adolescents: clinical outcome in adulthood. J Am Acad Child Adolesc Psychiatry.

[4] Andrews G, Issakidis C, Sanderson K, Corry J, Lapsley H (2004). Utilising survey data to inform public policy: comparison of the cost-effectiveness of treatment of ten mental disorders. Br J Psychiatry.

[5] Bor W, Dean AJ, Najman J, Hayatbakhsh R (2014). Are child and adolescent mental health problems increasing in the 21st century? A systematic review. Aust N Z J Psychiatry.

[6] Collishaw S, Maughan B, Natarajan L, Pickles A (2010). Trends in adolescent emotional problems in England: a comparison of two national cohorts twenty years apart. J Child Psychol Psychiatry.

[7] Rudolph KD, Lansford JE, Rodkin PC, Cicchetti D (2016). Interpersonal theories of developmental psychopathology. Developmental Psychopathology: Maladaptation and Psychopathology. Third Edition.

[8] Boomsma DI, van Beijsterveldt CE, Hudziak JJ (2005). Genetic and environmental influences on anxious/depression during childhood: a study from the Netherlands twin register. Genes Brain Behav.

[9] Eley TC, McAdams TA, Rijsdijk FV, Lichtenstein P, Narusyte J, Reiss D, Spotts EL, Ganiban JM, Neiderhiser JM (2015). The intergenerational transmission of anxiety: a children-of-twins study. Am J Psychiatry.

[10] Yap MB, Pilkington PD, Ryan SM, Jorm AF (2014). Parental factors associated with depression and anxiety in young people: a systematic review and meta-analysis. J Affect Disord.

[11] Pinquart M (2016). Associations of parenting dimensions and styles with internalizing symptoms in children and adolescents: a meta-analysis. Marriage Fam Rev.

[12] O'Connell ME, Boat T, Warner K (2009). Preventing Mental, Emotional, and Behavioral Disorders Among Young People: Progress and Possibilities.

[13] Cairns KE, Yap MB, Pilkington PD, Jorm AF (2014). Risk and protective factors for depression that adolescents can modify: a systematic review and meta-analysis of longitudinal studies. J Affect Disord.

[14] Beesdo K, Knappe S, Pine DS (2009). Anxiety and anxiety disorders in children and adolescents: developmental issues and implications for DSM-V. Psychiatr Clin North Am.

[15] Schleider JL, Weisz JR (2017). Family process and youth internalizing problems: a triadic model of etiology and intervention. Dev Psychopathol.

[16] Sameroff AJ (2009). The Transactional Model of Development: How Children and Contexts Shape Each Other.

[17] Sandler IN, Schoenfelder EN, Wolchik SA, MacKinnon DP (2011). Long-term impact of prevention programs to promote effective parenting: lasting effects but uncertain processes. Annu Rev Psychol.

[18] Yap MB, Morgan AJ, Cairns K, Jorm AF, Hetrick SE, Merry S (2016). Parents in prevention: a meta-analysis of randomized controlled trials of parenting interventions to prevent internalizing problems in children from birth to age 18. Clin Psychol Rev.

[19] Kehoe CE, Havighurst SS, Harley AE (2013). Tuning in to teens: improving parent emotion socialization to reduce youth internalizing difficulties. Soc Dev.

[20] Bearslee WR, Wright EJ, Gladstone TR, Forbes P (2007). Long-term effects from a randomized trial of two public health preventive interventions for parental depression. J Fam Psychol.

[21] Rotheram-Borus MJ, Stein JA, Lester P (2006). Adolescent adjustment over six years in HIV-affected families. J Adolesc Health.

[22] Sandler I, Ingram A, Wolchik S, Tein JY, Winslow E (2015). Long-term effects of parenting-focused preventive interventions to promote resilience of children and adolescents. Child Dev Perspect.

[23] Winslow EB, Bonds D, Wolchik S, Sandler I, Braver S (2009). Predictors of enrollment and retention in a preventive parenting intervention for divorced families. J Prim Prev.

[24] Morawska A, Sanders M (2006). A review of parental engagement in parenting interventions and strategies to promote it. J Child's Serv.

[25] Finan SJ, Swierzbiolek B, Priest N, Warren N, Yap M (2018). Parental engagement in preventive parenting programs for child mental health: a systematic review of predictors and strategies to increase engagement. PeerJ.

[26] Yap MB, Lawrence KA, Rapee RM, Cardamone-Breen MC, Green JM, Jorm AF (2017). Partners in parenting: a multi-level web-based approach to support parents in prevention and early intervention for adolescent depression and anxiety. JMIR Ment Health.

[27] Hansen A, Broomfield G, Yap MB (2019). A systematic review of technology-assisted parenting programs to prevent mental health problems in youth aged 0-18 years: applicability to underserved Australian communities. Aust J Psychol.

[28] Rutter M, Sroufe LA (2000). Developmental psychopathology: concepts and challenges. Dev Psychopathol.

[29] Mrazek PJ, Haggerty RJ (1994). Reducing Risks for Mental Disorders: Frontiers for Preventive Intervention Research.

[30] Oinas-Kukkonen H, Harjumaa M (2009). Persuasive systems design: key issues, process model, and system features. Commun Assoc Inf Syst.

[31] (2013). Parenting Strategies: Protecting Your Child's Mental Health.

[32] Yap MB, Pilkington PD, Ryan SM, Kelly CM, Jorm AF (2014). Parenting strategies for reducing the risk of adolescent depression and anxiety disorders: a Delphi consensus study. J Affect Disord.

[33] Sanders MR, Kirby JN (2012). Consumer engagement and the development, evaluation, and dissemination of evidence-based parenting programs. Behav Ther.

[34] Yap MB, Mahtani S, Rapee RM, Nicolas C, Lawrence KA, Mackinnon A, Jorm AF (2018). A tailored web-based intervention to improve parenting risk and protective factors for adolescent depression and anxiety problems: postintervention findings from a randomized controlled trial. J Med Internet Res.

[35] McClain DB, Wolchik SA, Winslow E, Tein JY, Sandler IN, Millsap RE (2010). Developmental cascade effects of the New Beginnings program on adolescent adaptation outcomes. Dev Psychopathol.

[36] Wolchik SA, Tein JY, Sandler IN, Kim HJ (2016). Developmental cascade models of a parenting-focused program for divorced families on mental health problems and substance use in emerging adulthood. Dev Psychopathol.

[37] Reuben JD, Shaw DS, Brennan LM, Dishion TJ, Wilson MN (2015). A family-based intervention for improving children's emotional problems through effects on maternal depressive symptoms. J Consult Clin Psychol.

[38] Cardamone-Breen MC, Jorm AF, Lawrence KA, Mackinnon AJ, Yap MB (2017). The parenting to reduce adolescent depression and anxiety scale: assessing parental concordance with parenting guidelines for the prevention of adolescent depression and anxiety disorders. PeerJ.

[39] (2015). Raising Children Network.

[40] Angold A, Costello E, Messer S, Pickles A, Winder F, Silver D (1995). Development of a short questionnaire for use in epidemiological studies of depression in children and adolescents. Int J Meth Psych Res.

[41] Rhew IC, Simpson K, Tracy M, Lymp J, McCauley E, Tsuang D, Stoep AV (2010). Criterion validity of the short mood and feelings questionnaire and one- and two-item depression screens in young adolescents. Child Adolesc Psychiatry Ment Health.

[42] Thapar A, McGuffin P (1998). Validity of the shortened mood and feelings questionnaire in a community sample of children and adolescents: a preliminary research note. Psychiatry Res.

[43] Nauta MH, Scholing A, Rapee RM, Abbott M, Spence SH, Waters A (2004). A parent-report measure of children's anxiety: psychometric properties and comparison with child-report in a clinic and normal sample. Behav Res Ther.

[44] Spence SH (1997). Structure of anxiety symptoms among children: a confirmatory factor-analytic study. J Abnorm Psychol.

[45] Spence SH, Barrett PM, Turner CM (2003). Psychometric properties of the Spence children's anxiety scale with young adolescents. J Anxiety Disord.

[46] Spence SH (1998). A measure of anxiety symptoms among children. Behav Res Ther.

[47] Kelders SM, Kok RN, Ossebaard HC, van Gemert-Pijnen JE (2012). Persuasive system design does matter: a systematic review of adherence to web-based interventions. J Med Internet Res.

[48] Spence SH (2016). Spence Children's Anxiety Scale.

[49] Bell ML, Fairclough DL (2014). Practical and statistical issues in missing data for longitudinal patient-reported outcomes. Stat Methods Med Res.

[50] Mallinckrodt CH, Sanger TM, Dubé S, DeBrota DJ, Molenberghs G, Carroll RJ, Potter WZ, Tollefson GD (2003). Assessing and interpreting treatment effects in longitudinal clinical trials with missing data. Biol Psychiatry.

[51] Gueorguieva R, Krystal JH (2004). Move over ANOVA: progress in analyzing repeated-measures data and its reflection in papers published in the archives of general psychiatry. Arch Gen Psychiatry.

[52] Hayes AF (2018). PROCESS Macro.

[53] Hayes AF (2018). Introduction to Mediation, Moderation, and Conditional Process Analysis: A Regression-Based Approach. Second Edition.

[54] Hetrick SE, Cox GR, Witt KG, Bir JJ, Merry SN (2016). Cognitive behavioural therapy (CBT), third-wave CBT and interpersonal therapy (IPT) based interventions for preventing depression in children and adolescents. Cochrane Database Syst Rev.

[55] Rose G (1992). Rose's Strategy of Preventive Medicine.

[56] Branje SJ, Laursen B, Collins WA, Vangelisti AL (2012). Parent-child communication during adolescence. The Routledge Handbook of Family Communication. Second Edition.

[57] Collins WA, Laursen B, Hendrick SS, Hendrick C (2000). Adolescent relationships: the art of fugue. Close Relationships: A Sourcebook.

[58] de los Reyes A, Ohannessian CM, Racz SJ (2019). Discrepancies between adolescent and parent reports about family relationships. Child Dev Perspect.

[59] Yap MB (2018). Treatment of paediatric anxiety disorders: what next?. Lancet Child Adolesc Health.

[60] Frank TJ, Keown LJ, Sanders MR (2015). Enhancing father engagement and interparental teamwork in an evidence-based parenting intervention: a randomized-controlled trial of outcomes and processes. Behav Ther.

[61] Reavley NJ, Jorm AF (2014). Mental health reform: increased resources but limited gains. Med J Aust.

