# Figure Correction: Medium-Term Effects of a Tailored Web-Based Parenting Intervention to Reduce Adolescent Risk of Depression and Anxiety: 12-Month Findings From a Randomized Controlled Trial

**DOI:** 10.2196/15915

**Published:** 2019-08-29

**Authors:** Marie Bee Hui Yap, Mairead C Cardamone-Breen, Ronald M Rapee, Katherine A Lawrence, Andrew J Mackinnon, Shireen Mahtani, Anthony F Jorm

**Affiliations:** 1 School of Psychological Sciences and Turner Institute for Brain and Mental Health Monash University Melbourne Australia; 2 Melbourne School of Population and Global Health University of Melbourne Melbourne Australia; 3 Centre for Emotional Health Macquarie University Sydney Australia; 4 Black Dog Institute University of New South Wales Sydney Australia

In “Medium-Term Effects of a Tailored Web-Based Parenting Intervention to Reduce Adolescent Risk of Depression and Anxiety: 12-Month Findings From a Randomized Controlled Trial” (J Med Internet Res 2019;21(8):e13628), Figure 2 was erroneously published with one graph missing from the image. The graph of Parenting to Reduce Adolescent Depression and Anxiety Scale (PRADAS) was not shown. The image has now been replaced with a version that includes two graphs:

1. Estimated marginal means for Parenting to Reduce Adolescent Depression and Anxiety Scale (PRADAS)
2. Estimated marginal means for Parenting to Reduce Adolescent Depression and Anxiety Scale—Adolescent report (PRADAS-A)

The correction will appear in the online version of the paper on the JMIR website on August 29, 2019, together with the publication of this correction notice. Because this was made after submission to PubMed, PubMed Central, and other full-text repositories, the corrected article also has been resubmitted to those repositories.

**Figure 2:**
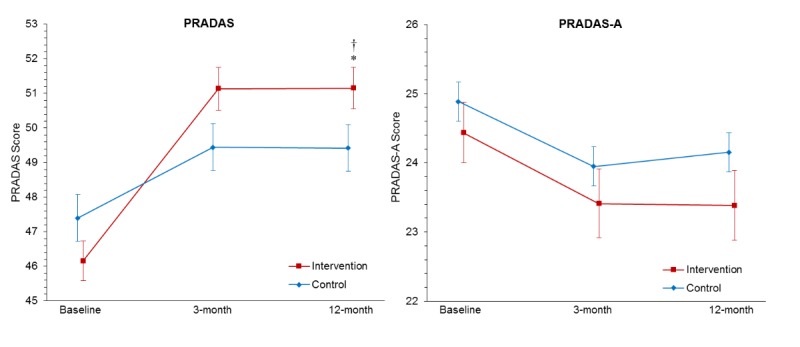
Estimated marginal means for Parenting to Reduce Adolescent Depression and Anxiety Scale (PRADAS) and PRADAS—Adolescent (PRADAS-A) Report scores at baseline, postintervention (3-months postbaseline), and 12-month follow-up, estimated under the group × measurement-occasion mixed model. Error bars represent SEs. Higher scores on the PRADAS and PRADAS—Adolescent Report indicate greater concordance with the parenting guidelines (ie, more protective parenting factors and fewer parenting risk factors). Planned contrast of interaction (baseline to 12 months) was significant, *P*<.001. Pairwise comparison of group difference at 12-month follow-up was significant, *P*=.04.

