# Supplementary material for: Medium-Term Effects of a Tailored Web-Based Parenting Intervention to Reduce Adolescent Risk of Depression and Anxiety: 12-Month Findings From a Randomized Controlled Trial
Source: J Med Internet Res. 2019 Aug 15;21(8):e13628. doi: 10.2196/13628 (PMC6830751; doi:10.2196/13628)
Supplement: Multimedia Appendix 3 [file jmir_v21i8e13628_app3.pdf]

Multimedia Appendix 3: Sample characteristics at baseline

Table 1

Characteristics of the parent participant sample at baseline by intervention arm

	Intervention (n = 179)	Control (n = 180)	t or χ^2	p
Parent gender			0.94[a]	.33[a]
Male, n (%)	26 (14.5)	20 (11.1)		
Female, n (%)	153 (85.5)	160 (88.9)		
Parent age, M (SD)	45.2 (5.26)	45.1 (5.14)	0.14	.89
Parent marital status			3.23	.36
Single, n (%)	12 (6.7)	9 (5.0)		
Married/de facto, n (%)	138 (77.1)	137 (76.1)		
Separated/divorced, n (%)	27 (15.1)	34 (18.9)		
Widowed, n (%)	2 (1.1)	0 (0)		
Child gender			0.35[a]	.56[a]
Male, n (%)	102 (57.0)	97 (53.9)		
Female, n (%)	77 (43.0)	83 (46.1)		
Child age, M (SD)	13.7 (1.05)	13.7 (1.08)	-0.43	.67
Family situation			8.77	.07
Child participant lives with both parents, n (%)	131 (73.2)	122 (67.8)		
Parents separated but both involved in care of child participant, n (%)	21 (11.7)	36 (20.0)		
Parents separated with only registered parent involved in care of child participant, n (%)	16 (8.9)	14 (7.8)		
Sole parent of child participant, n (%)	10 (5.6)	4 (2.2)		
Other, n (%)	1 (0.6)	4 (2.2)		
Number of children, M (SD)	2.37 (0.94)	2.32 (1.00)	0.45	.65
Language			0.03[a]	.87[a]
English, n (%)	150 (83.8)	152 (84.4)		
Other, n (%)	29 (16.2)	28 (15.6)		
Parent employment			1.56[a]	.46
Unemployed, n (%)	21 (11.7)	27 (15.0)		
Part-time, n (%)	81 (45.3)	71 (39.4)		
Full-time, n (%)	77 (43.0)	82 (45.6)		
Parent studying status			1.55	.46
Not studying, n (%)	149 (83.2)	145 (80.6)		
Studying part-time, n (%)	4 (2.2)	2 (1.1)		

Studying full-time, *n* (%)	26 (14.5)	33 (18.3)		
Parent's highest education level			3.93[a]	.56[a]
Year 7-12, *n* (%)	26 (14.5)	24 (13.3)		
Trade/apprenticeship, *n* (%)	2 (1.1)	4 (2.2)		
Other TAFE/Technical, *n* (%)	18 (10.1)	13 (6.7)		
Diploma, *n* (%)	26 (14.5)	37 (21.1)		
Bachelor degree, *n* (%)	63 (35.2)	56 (31.1)		
Postgraduate degree, *n* (%)	44 (24.6)	46 (25.6)		
Parent's mental health diagnosis			5.11[a]	.16[a]
None, *n* (%)	72 (40.2)	72 (40.0)		
Past history, *n* (%)	60 (33.5)	77 (42.8) [p]		
Current diagnosis, *n* (%)	25 (14.0)	17 (9.4)		
Past and current diagnosis, *n* (%)	20 (11.2)	13 (7.2)		
Unanswered, *n* (%)	2 (1.1)	1 (0.6)		
Child's past mental health diagnosis			9.26[a]	.24[a]
Depression, *n* (%)	3 (1.7)	0 (0)		
Any anxiety disorder, *n* (%)	12 (6.7)	13 (7.2)		
Depression and anxiety	1 (0.6)	1 (1.0)		
Autism or Asperger's syndrome, *n* (%)	4 (2.2)	5 (2.8)		
Other, *n* (%)	11 (6.1)	20 (11.1)		
Multiple diagnoses, *n* (%)	5 (2.8)	8 (4.4)		
No formal diagnosis, but parent concerned, *n* (%)	26 (14.5)	43 (23.9)		
No diagnosis, *n* (%)	105 (58.7)	87 (48.3)		
Unanswered, *n* (%)	16 (8.9)	14 (7.8)		
Child's current mental health diagnosis			5.51[a]	.60[a]
Depression, *n* (%)	0 (0)	1 (0.5)		
Any anxiety disorder, *n* (%)	14 (15.5)	17 (15.5)		
Depression and anxiety	3 (2.5)	2 (1.1)		
Autism or Asperger's syndrome[b], *n* (%)	3 (1.7)	4 (2.2)		
Other, *n* (%)	11 (9.5)	8 (9.5)		
Multiple diagnoses, *n* (%)	8 (9.0)	10 (5.6)		
No formal diagnosis, but parent concerned, *n* (%)	36 (20.1)	50 (27.8)		
No diagnosis, *n* (%)	104 (58.1)	88 (48.9)		
Unanswered, *n* (%)	3 (1.7)	4 (2.2)		

[a] Values differ to original publication (Yap et al., 2018) due to errors detected when preparing the current paper.
[b]Two children who were reported by their parents to have a past diagnosis of autism or Asperger's syndrome were categorized under "Multiple diagnosis" as they also had another current mental health diagnosis.
Adapted from: Yap, M. B. H., Mahtani, S., Rapee, R. M., Nicolas, C., Lawrence, K. A., Mackinnon, A. & Jorm, A. F. (2018). A tailored web-based intervention to improve parenting risk and protective factors for adolescent

depression and anxiety problems: Postintervention findings from a randomized controlled trial. *Journal of Medical Internet Research, 20*(1), e17. doi:10.2196/jmir.9139
