# Supplementary material for: Medium-Term Effects of a Tailored Web-Based Parenting Intervention to Reduce Adolescent Risk of Depression and Anxiety: 12-Month Findings From a Randomized Controlled Trial
Source: J Med Internet Res. 2019 Aug 15;21(8):e13628. doi: 10.2196/13628 (PMC6830751; doi:10.2196/13628)
Supplement: Multimedia Appendix 4 [file jmir_v21i8e13628_app4.pdf]

Multimedia Appendix 4: Observed scores for all outcome measures at each measurement occasion

Table 1

Means, standard deviations and sample size for all outcome measures at each measurement occasion, by intervention arm

Outcome	Group	
	Intervention	Control
	M (SD), n	*M (SD), n*
**PRADAS**		
Baseline	46.58 (7.53)<br>*n* = 179	47.88 (7.60)<br>*n* = 180
Post-intervention (3 months post baseline)	52.06 (8.42)<br>*n* = 155	49.96 (7.55)<br>*n* = 164
12-month follow-up	51.81 (7.92)<br>*n* = 158	50.21 (7.52)<br>*n* = 159
**PRADAS-A**		
Baseline	24.44 (5.62)<br>*n* = 163	24.91 (5.68)<br>*n* = 169
Post-intervention	23.38 (6.35)<br>*n* = 150	24.09(6.20)<br>*n* = 158
12-month follow-up	23.53 (6.26)<br>*n* = 141	24.32 (6.28)<br>*n* = 146
**SCAS-P**		
Baseline	17.99 (11.60)<br>*n* = 179	18.51 (12.36)<br>*n* = 180
Post-intervention	15.19 (12.10)<br>*n* = 154	14.78 (11.07)<br>*n* = 164
12-month follow-up	13.95 (11.74)<br>*n* = 155	15.42 (12.79)<br>*n* = 159
**SCAS-C**		
Baseline	28.73 (17.34)<br>*n* = 163	30.14 (17.40)<br>*n* = 169
Post-intervention	28.31 (20.22)<br>*n* = 150	26.70 (17.11)<br>*n* = 158
12-month follow-up	26.36 (18.97)<br>*n* = 141	25.64 (16.93)<br>*n* = 145
**SMFQ-P**		
Baseline	5.07 (5.62)<br>*n* = 179	4.75 (4.98)<br>*n* = 180
Post-intervention	3.60 (4.32)<br>*n* = 154	3.38 (4.38)<br>*n* = 164
12-month follow-up	3.46 (5.15)<br>*n* = 155	4.16 (5.09)<br>*n* = 159
**SMFQ-C**		
Baseline	6.16 (6.12)<br>*n* = 163	6.42 (5.79)<br>*n* = 169
Post-intervention	6.25 (6.60)<br>*n* = 150	6.03 (5.49)<br>*n* = 158
12-month follow-up	6.70 (6.64)<br>*n* = 141	6.75 (6.91)<br>*n* = 145
