# Supplementary material for: Medium-Term Effects of a Tailored Web-Based Parenting Intervention to Reduce Adolescent Risk of Depression and Anxiety: 12-Month Findings From a Randomized Controlled Trial
Source: J Med Internet Res. 2019 Aug 15;21(8):e13628. doi: 10.2196/13628 (PMC6830751; doi:10.2196/13628)
Supplement: Multimedia Appendix 5 [file jmir_v21i8e13628_app5.pdf]

Multimedia Appendix 5: Results of primary and secondary outcome MRRMs, run on square root transformed data

Table 1

MMRM planned contrast test of baseline to 12-month interaction, for square root transformed symptom measures

Outcome measure	Estimated Marginal Means (SE)		t^a	df	*p*	$d_{interaction}$ (95% CI)[b]	$d_{12\text{-month}}$ (95% CI)[c]
	Intervention	Control					
**SCAS-P**			-1.18	319.21	.237	-0.13 (-0.33, 0.08)	-0.11 (-0.33, 0.11)
Baseline	4.00 (0.10)	4.08 (0.10)					
12-month follow-up	3.42 (0.12)	3.65 (0.11)					
**SCAS-C**			1.17	296.14	.245	0.13 (-0.09, 0.34)	0.00 (-0.23, 0.24)
Baseline	5.13 (0.12)	5.29 (0.12)					
12-month follow-up	4.88 (0.14)	4.87 (0.14)					
**SMFQ-P**			-2.04	324.98	.043	-0.21 (-0.42, -0.01)	-0.17 (-0.39, 0.05)
Baseline	1.85 (0.09)	1.81 (0.09)					
12-month follow-up	1.34 (0.10)	1.58 (0.10)					
**SMFQ-C**			0.75	300.80	.454	0.08 (-0.13, 0.30)	0.03 (-0.20, 0.26)
Baseline	2.16 (0.10)	2.21 (0.09)					
12-month follow-up	2.25 (0.12)	2.19 (0.12)					

[a]*t* statistic of the planned contrast test of baseline to 12-month interaction, estimated under the group × measurement occasion mixed model.
[b]Cohen's *d* effect size of the baseline to 12-month interaction effect, calculated based on the *t* statistic of the planned contrast.
[c]Cohen's *d* effect size of the difference between groups at 12-month follow-up.
