# Supplementary material for: Medium-Term Effects of a Tailored Web-Based Parenting Intervention to Reduce Adolescent Risk of Depression and Anxiety: 12-Month Findings From a Randomized Controlled Trial
Source: J Med Internet Res. 2019 Aug 15;21(8):e13628. doi: 10.2196/13628 (PMC6830751; doi:10.2196/13628)
Supplement: Multimedia Appendix 6 [file jmir_v21i8e13628_app6.pdf]

Multimedia Appendix 6: Results of mediation analyses

Table 1

Indirect effects of group on 12-month symptom measures scores, mediated by post-intervention PRADAS score.

Outcome measure[a]	*b*	Boot SE	95% CI[b]
SCAS-P	0.24	0.35	-0.42, 0.95
SCAS-C	0.19	0.45	-0.62, 1.17
SMFQ-P	-0.26	0.15	-0.57, 0.03
SMFQ-C	-0.12	0.20	-0.53, 0.27

[a]All models include post-intervention PRADAS score as the mediator variable and baseline PRADAS and baseline symptom measure (corresponding to the outcome measure) as covariates.
[b]Confidence intervals are bias-corrected bootstrapped 95% confidence intervals based on 5000 bootstrapped samples.

Table 2

Indirect effects of group on 12-month symptom measures scores, mediated by post-intervention PRADAS score, using square root transformed data for symptom measures.

Outcome measure[a, b]	*b*	Boot SE	95% CI[c]
SCAS-P	0.02	0.04	-0.06, 0.10
SCAS-C	0.03	0.04	-0.05, 0.12
SMFQ-P	-0.08	0.04	-0.16, -0.01
SMFQ-C	-0.01	0.04	-0.09, 0.07

[a]All models include post-intervention PRADAS score as the mediator variable and baseline PRADAS and baseline symptom measure (corresponding to the outcome measure) as covariates.
[b]All symptom measure variables were square root transformed.
[c]Confidence intervals are bias-corrected bootstrapped 95% confidence intervals based on 5000 bootstrapped samples.
