# Supplementary material for: Medium-Term Effects of a Tailored Web-Based Parenting Intervention to Reduce Adolescent Risk of Depression and Anxiety: 12-Month Findings From a Randomized Controlled Trial
Source: J Med Internet Res. 2019 Aug 15;21(8):e13628. doi: 10.2196/13628 (PMC6830751; doi:10.2196/13628)
Supplement: Multimedia Appendix 7 [file jmir_v21i8e13628_app7.pdf]

Multimedia Appendix 7: Results of *post-hoc* moderation analyses

Table 1

MMRM three-way interaction results of primary and secondary outcomes moderated by baseline symptom measures

Outcome measure and moderator variable	F^a	df	p	$d_{\text{interaction}}$ (95% CI)[b]
**PRADAS**				
Moderated by SCAS-C	1.79	2, 296.73	.168	0.15 (-0.07, 0.36)
Moderated by SMFQ-C	0.04	2, 295.71	.964	0.02 (-0.19, 0.24)
**PRADAS-A**				
Moderated by SCAS-P	10.23	2, 296.81	< .001	0.35 (0.13, 0.57)
Moderated by SMFQ-P	2.02	2, 297.56	.13	0.16 (-0.06, 0.37)
**SCAS-P**				
Moderated by SCAS-C	0.13	2, 287.16	.878	0.04 (-0.18, 0.26)
**SCAS-C**				
Moderated by SCAS-P	2.19	2, 296.68	.113	0.16 (-0.05, 0.38)
**SMFQ-P**				
Moderated by SMFQ-C	4.40	2, 295.27	.013	0.23 (0.01, 0.45)
**SMFQ-C**				
Moderated by SMFQ-P	0.33	2, 295.88	.718	0.06 (-0.15, 0.28)

[a]F test of the three-way group × measurement occasion × moderator interaction, estimated under group × measurement occasion mixed model.
[b]Cohen's *d* effect size of the group × measurement occasion × moderator interaction effect.

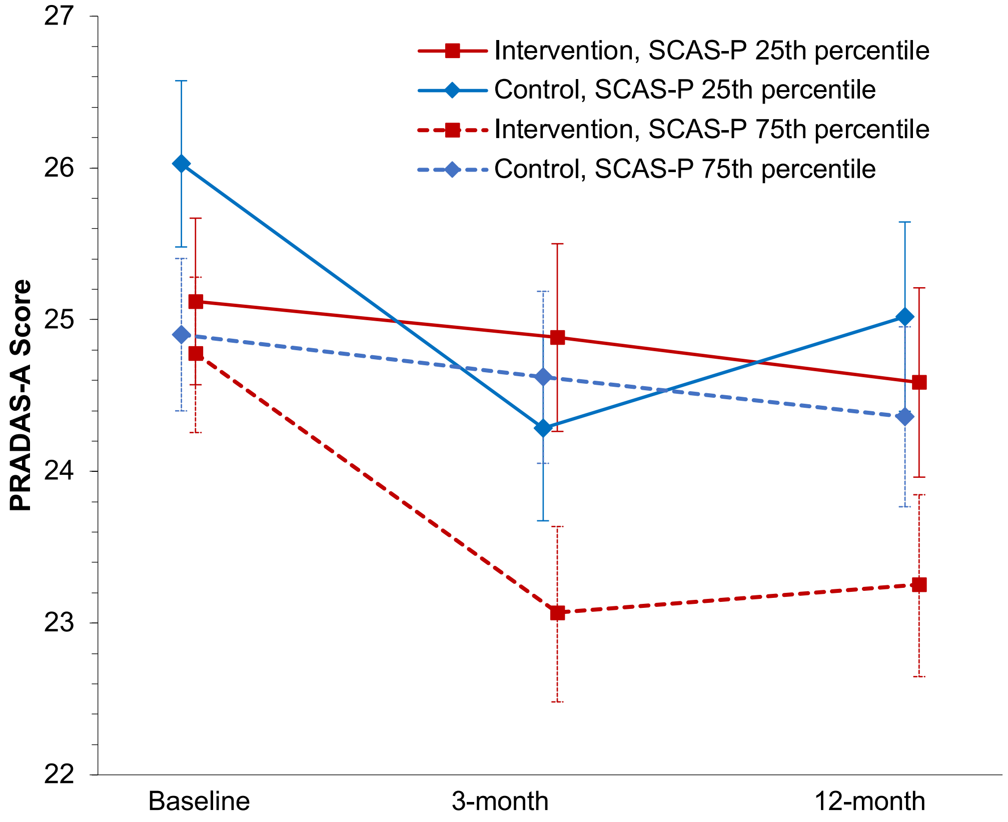

Figure 1. Estimated marginal means of PRADAS-A, with baseline SCAS-P (moderator variable) calculated at the 25th and 75th percentiles. Estimated under the group × measurement occasion × moderator mixed model. Error bars represent standard error.
25th percentile baseline SCAS-P = 9; 75th percentile baseline SCAS-P = 25.

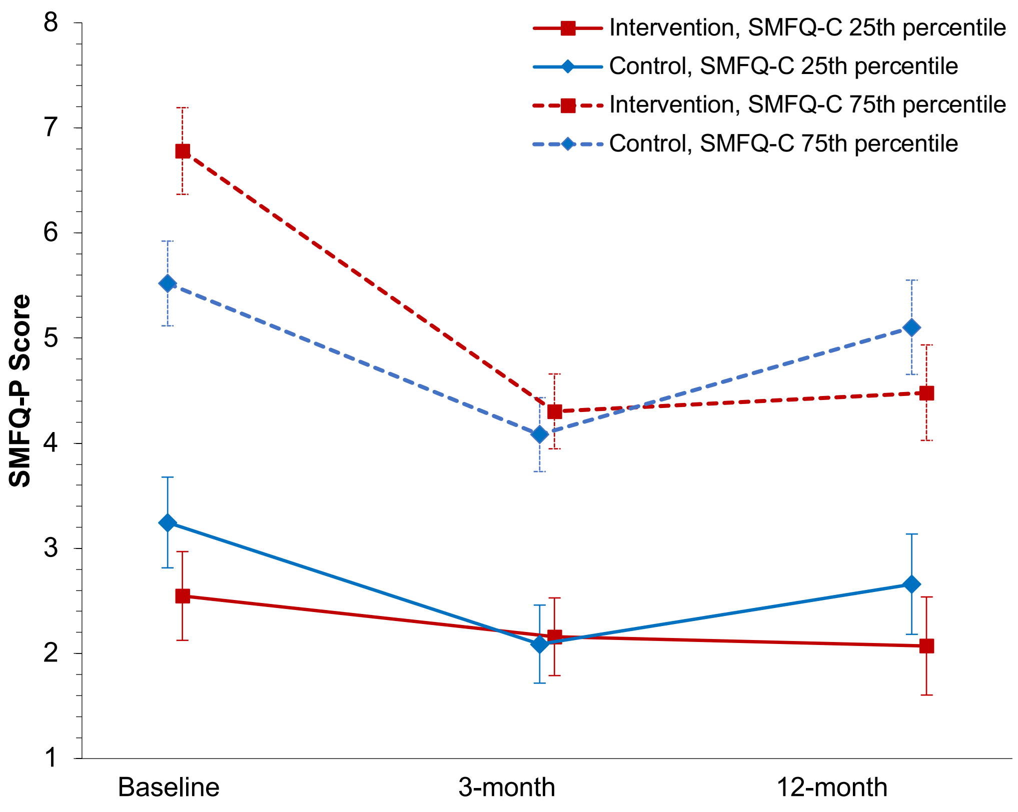

Figure 2. Estimated marginal means of SMFQ-P, with baseline SMFQ-C (moderator variable) calculated at the 25th and 75th percentiles. Estimated under the group × measurement occasion × moderator mixed model. Error bars represent standard error.
25th percentile baseline SMFQ-C = 2; 75th percentile baseline SMFQ-C = 10.
