# Supplementary material for: Medium-Term Effects of a Tailored Web-Based Parenting Intervention to Reduce Adolescent Risk of Depression and Anxiety: 12-Month Findings From a Randomized Controlled Trial
Source: J Med Internet Res. 2019 Aug 15;21(8):e13628. doi: 10.2196/13628 (PMC6830751; doi:10.2196/13628)
Supplement: Multimedia Appendix 8 [file jmir_v21i8e13628_app8.pdf]

CONSORT-EHEALTH (V 1.6.1) - Submission/Publication Form

The CONSORT-EHEALTH checklist is intended for authors of randomized trials evaluating web-based and Internet-based applications/interventions, including mobile interventions, electronic games (incl multiplayer games), social media, certain telehealth applications, and other interactive and/or networked electronic applications. Some of the items (e.g. all subitems under item 5 - description of the intervention) may also be applicable for other study designs.

The goal of the CONSORT EHEALTH checklist and guideline is to be
a) a guide for reporting for authors of RCTs,
b) to form a basis for appraisal of an ehealth trial (in terms of validity)

CONSORT-EHEALTH items/subitems are MANDATORY reporting items for studies published in the Journal of Medical Internet Research and other journals / scientific societies endorsing the checklist.

Items numbered 1., 2., 3., 4a., 4b etc are original CONSORT or CONSORT-NPT (non-pharmacologic treatment) items.
Items with Roman numerals (i., ii, iii, iv etc.) are CONSORT-EHEALTH extensions/clarifications.

As the CONSORT-EHEALTH checklist is still considered in a formative stage, we would ask that you also RATE ON A SCALE OF 1-5 how important/useful you feel each item is FOR THE PURPOSE OF THE CHECKLIST and reporting guideline (optional).

Mandatory reporting items are marked with a red *.
In the textboxes, either copy & paste the relevant sections from your manuscript into this form - please include any quotes from your manuscript in QUOTATION MARKS,
or answer directly by providing additional information not in the manuscript, or elaborating on why the item was not relevant for this study.

YOUR ANSWERS WILL BE PUBLISHED AS A SUPPLEMENTARY FILE TO YOUR PUBLICATION IN JMIR AND ARE CONSIDERED PART OF YOUR PUBLICATION (IF ACCEPTED).
Please fill in these questions diligently. Information will not be copyedited, so please use proper spelling and grammar, use correct capitalization, and avoid abbreviations.

DO NOT FORGET TO SAVE AS PDF _AND_ CLICK THE SUBMIT BUTTON SO YOUR ANSWERS ARE IN OUR DATABASE !!!

Citation Suggestion (if you append the pdf as Appendix we suggest to cite this paper in the caption):
Eysenbach G, CONSORT-EHEALTH Group
CONSORT-EHEALTH: Improving and Standardizing Evaluation Reports of Web-based and Mobile Health Interventions
J Med Internet Res 2011;13(4):e126
URL: http://www.jmir.org/2011/4/e126/
doi: 10.2196/jmir.1923
PMID: 22209829

* Required

Your name *

First Last

Mairead Cardamone-Breen

Primary Affiliation (short), City, Country *

University of Toronto, Toronto, Canada

Monash University, Melbourne, Australia

Your e-mail address *

abc@gmail.com

mairead.cardamone-breen@monash.edu

Title of your manuscript *

Provide the (draft) title of your manuscript.

A Tailored Web-Based Parenting Intervention to Reduce Adolescent Risk for Depression and Anxiety: 12-month Follow-Up Findings from a Randomised Controlled Trial

Name of your App/Software/Intervention *

If there is a short and a long/alternate name, write the short name first and add the long name in brackets.

Partners in Parenting (PiP)

Evaluated Version (if any)

e.g. "V1", "Release 2017-03-01", "Version 2.0.27913"

Your answer

Language(s) *

What language is the intervention/app in? If multiple languages are available, separate by comma (e.g. "English, French")

English

URL of your Intervention Website or App

e.g. a direct link to the mobile app on app in appstore (itunes, Google Play), or URL of the website. If the intervention is a DVD or hardware, you can also link to an Amazon page.

Your answer

URL of an image/screenshot (optional)

Your answer

Accessibility *

Can an enduser access the intervention presently?

- ○ access is free and open
- ○ access only for special usergroups, not open
- ○ access is open to everyone, but requires payment/subscription/in-app purchases
- ○ app/intervention no longer accessible
- ◉ Other: Not currently publicly available.

Primary Medical Indication/Disease/Condition *

e.g. "Stress", "Diabetes", or define the target group in brackets after the condition, e.g. "Autism (Parents of children with)", "Alzheimers (Informal Caregivers of)"

Depression and anxiety (adolescents)

Primary Outcomes measured in trial *

comma-separated list of primary outcomes reported in the trial

Parent-report parenting risk and protecti

Secondary/other outcomes

Are there any other outcomes the intervention is expected to affect?

Adolescent-reported parenting (risk and protective factors for adolescent depression and anxiety)

Recommended "Dose" *

What do the instructions for users say on how often the app should be used?

- ( ) Approximately Daily
- (•) Approximately Weekly
- ( ) Approximately Monthly
- ( ) Approximately Yearly
- ( ) "as needed"
- ( ) Other:

Approx. Percentage of Users (starters) still using the app as recommended after 3 months *

- (x) unknown / not evaluated
- ( ) 0-10%
- ( ) 11-20%
- ( ) 21-30%
- ( ) 31-40%
- ( ) 41-50%
- ( ) 51-60%
- ( ) 61-70%
- ( ) 71%-80%
- ( ) 81-90%
- ( ) 91-100%
- ( ) Other:

Overall, was the app/intervention effective? *

- ( ) yes: all primary outcomes were significantly better in intervention group vs control
- (x) partly: SOME primary outcomes were significantly better in intervention group vs control
- ( ) no statistically significant difference between control and intervention
- ( ) potentially harmful: control was significantly better than intervention in one or more outcomes
- ( ) inconclusive: more research is needed
- ( ) Other:

Article Preparation Status/Stage *

At which stage in your article preparation are you currently (at the time you fill in this form)

- ○ not submitted yet - in early draft status
- ◉ not submitted yet - in late draft status, just before submission
- ○ submitted to a journal but not reviewed yet
- ○ submitted to a journal and after receiving initial reviewer comments
- ○ submitted to a journal and accepted, but not published yet
- ○ published
- ○ Other:

Journal *

If you already know where you will submit this paper (or if it is already submitted), please provide the journal name (if it is not JMIR, provide the journal name under "other")

- ○ not submitted yet / unclear where I will submit this
- ◉ Journal of Medical Internet Research (JMIR)
- ○ JMIR mHealth and UHealth
- ○ JMIR Serious Games
- ○ JMIR Mental Health
- ○ JMIR Public Health
- ○ JMIR Formative Research
- ○ Other JMIR sister journal
- ○ Other:

Is this a full powered effectiveness trial or a pilot/feasibility trial? *

- ○ Pilot/feasibility
- ◉ Fully powered

Manuscript tracking number *

If this is a JMIR submission, please provide the manuscript tracking number under "other" (The ms tracking number can be found in the submission acknowledgement email, or when you login as author in JMIR. If the paper is already published in JMIR, then the ms tracking number is the four-digit number at the end of the DOI, to be found at the bottom of each published article in JMIR)

- (selected) no ms number (yet) / not (yet) submitted to / published in JMIR
- Other:

TITLE AND ABSTRACT

1a) TITLE: Identification as a randomized trial in the title

1a) Does your paper address CONSORT item 1a? *

I.e does the title contain the phrase "Randomized Controlled Trial"? (if not, explain the reason under "other")

- (selected) yes
- Other:

1a-i) Identify the mode of delivery in the title

Identify the mode of delivery. Preferably use "web-based" and/or "mobile" and/or "electronic game" in the title. Avoid ambiguous terms like "online", "virtual", "interactive". Use "Internet-based" only if Intervention includes non-web-based Internet components (e.g. email), use "computer-based" or "electronic" only if offline products are used. Use "virtual" only in the context of "virtual reality" (3-D worlds). Use "online" only in the context of "online support groups". Complement or substitute product names with broader terms for the class of products (such as "mobile" or "smart phone" instead of "iphone"), especially if the application runs on different platforms.

	1	2	3	4	5	
subitem not at all important	○	○	○	○	●	essential

Does your paper address subitem 1a-i? *

Copy and paste relevant sections from manuscript title (include quotes in quotation marks "like this" to indicate direct quotes from your manuscript), or elaborate on this item by providing additional information not in the ms, or briefly explain why the item is not applicable/relevant for your study

"A Tailored Web-Based Parenting Intervention to Reduce Adolescent Risk for Depression and Anxiety: 12-month Follow-Up Findings from aA Randomised Controlled Trial"

1a-ii) Non-web-based components or important co-interventions in title

Mention non-web-based components or important co-interventions in title, if any (e.g., “with telephone support”).

	1	2	3	4	5	
subitem not at all important	○	○	○	◉	○	essential

Does your paper address subitem 1a-ii?

Copy and paste relevant sections from manuscript title (include quotes in quotation marks "like this" to indicate direct quotes from your manuscript), or elaborate on this item by providing additional information not in the ms, or briefly explain why the item is not applicable/relevant for your study

"A Tailored Web-Based Parenting Intervention to Reduce Adolescent Risk for Depression and Anxiety: 12-month Follow-Up Findings from aA Randomised Controlled Trial"

1a-iii) Primary condition or target group in the title

Mention primary condition or target group in the title, if any (e.g., “for children with Type I Diabetes”) Example: A Web-based and Mobile Intervention with Telephone Support for Children with Type I Diabetes: Randomized Controlled Trial

	1	2	3	4	5	
subitem not at all important	○	○	○	○	◉	essential

Does your paper address subitem 1a-iii? *

Copy and paste relevant sections from manuscript title (include quotes in quotation marks "like this" to indicate direct quotes from your manuscript), or elaborate on this item by providing additional information not in the ms, or briefly explain why the item is not applicable/relevant for your study

"A Tailored Web-Based Parenting Intervention to Reduce Adolescent Risk for Depression and Anxiety: 12-month Follow-Up Findings from aA Randomised Controlled Trial"

1b) ABSTRACT: Structured summary of trial design, methods, results, and conclusions

NPT extension: Description of experimental treatment, comparator, care providers, centers, and blinding status.

1b-i) Key features/functionalities/components of the intervention and comparator in the METHODS section of the ABSTRACT

Mention key features/functionalities/components of the intervention and comparator in the abstract. If possible, also mention theories and principles used for designing the site. Keep in mind the needs of systematic reviewers and indexers by including important synonyms. (Note: Only report in the abstract what the main paper is reporting. If this information is missing from the main body of text, consider adding it)

	1	2	3	4	5	
subitem not at all important	○	○	○	◉	○	essential

Does your paper address subitem 1b-i? *

Copy and paste relevant sections from the manuscript abstract (include quotes in quotation marks "like this" to indicate direct quotes from your manuscript), or elaborate on this item by providing additional information not in the ms, or briefly explain why the item is not applicable/relevant for your study

"Parents in the PiP intervention condition received personalized feedback about their parenting and were recommended a series of up to nine interactive modules. Control group parents received access to five educational factsheets about adolescent development and mental health. Both groups received a weekly 5-minute phone call to encourage progress through their program."

1b-ii) Level of human involvement in the METHODS section of the ABSTRACT

Clarify the level of human involvement in the abstract, e.g., use phrases like "fully automated" vs. "therapist/nurse/care provider/physician-assisted" (mention number and expertise of providers involved, if any). (Note: Only report in the abstract what the main paper is reporting. If this information is missing from the main body of text, consider adding it)

	1	2	3	4	5	
subitem not at all important	○	○	○	◉	○	essential

Does your paper address subitem 1b-ii?

Copy and paste relevant sections from the manuscript abstract (include quotes in quotation marks "like this" to indicate direct quotes from your manuscript), or elaborate on this item by providing additional information not in the ms, or briefly explain why the item is not applicable/relevant for your study

"Both groups received a weekly 5-minute phone call to encourage progress through their program."

1b-iii) Open vs. closed, web-based (self-assessment) vs. face-to-face assessments in the METHODS section of the ABSTRACT

Mention how participants were recruited (online vs. offline), e.g., from an open access website or from a clinic or a closed online user group (closed usergroup trial), and clarify if this was a purely web-based trial, or there were face-to-face components (as part of the intervention or for assessment). Clearly say if outcomes were self-assessed through questionnaires (as common in web-based trials). Note: In traditional offline trials, an open trial (open-label trial) is a type of clinical trial in which both the researchers and participants know which treatment is being administered. To avoid confusion, use “blinded” or “unblinded” to indicated the level of blinding instead of “open”, as “open” in web-based trials usually refers to “open access” (i.e. participants can self-enrol). (Note: Only report in the abstract what the main paper is reporting. If this information is missing from the main body of text, consider adding it)

	1	2	3	4	5	
subitem not at all important	○	○	◉	○	○	essential

Does your paper address subitem 1b-iii?

Copy and paste relevant sections from the manuscript abstract (include quotes in quotation marks "like this" to indicate direct quotes from your manuscript), or elaborate on this item by providing additional information not in the ms, or briefly explain why the item is not applicable/relevant for your study

"Parents (n = 359) and adolescents (n = 332) were recruited primarily from secondary schools and completed online assessments of parenting and adolescent depression and anxiety symptoms at baseline, post-intervention (3 months later), and 12-month follow-up (n = 317 parents, 287 adolescents)."

1b-iv) RESULTS section in abstract must contain use data

Report number of participants enrolled/assessed in each group, the use/uptake of the intervention (e.g., attrition/adherence metrics, use over time, number of logins etc.), in addition to primary/secondary outcomes. (Note: Only report in the abstract what the main paper is reporting. If this information is missing from the main body of text, consider adding it)

	1	2	3	4	5	
subitem not at all important	○	○	◉	○	○	essential

Does your paper address subitem 1b-iv?

Copy and paste relevant sections from the manuscript abstract (include quotes in quotation marks "like this" to indicate direct quotes from your manuscript), or elaborate on this item by providing additional information not in the ms, or briefly explain why the item is not applicable/relevant for your study

" Intervention group parents completed an average of 73.7% of their intended program."

1b-v) CONCLUSIONS/DISCUSSION in abstract for negative trials

Conclusions/Discussions in abstract for negative trials: Discuss the primary outcome - if the trial is negative (primary outcome not changed), and the intervention was not used, discuss whether negative results are attributable to lack of uptake and discuss reasons. (Note: Only report in the abstract what the main paper is reporting. If this information is missing from the main body of text, consider adding it)

	1	2	3	4	5	
subitem not at all important	○	○	◉	○	○	essential

Does your paper address subitem 1b-v?

Copy and paste relevant sections from the manuscript abstract (include quotes in quotation marks "like this" to indicate direct quotes from your manuscript), or elaborate on this item by providing additional information not in the ms, or briefly explain why the item is not applicable/relevant for your study

Not applicable, the primary outcome was not negative. Negative results for secondary outcomes are reported in the abstract:
"For the primary outcome of parent-reported parenting, the intervention group showed significantly greater improvement from baseline to 12-month follow-up compared to controls, with a medium effect size (Cohen's d = 0.51; 95% CI 0.30, 0.72). No significant intervention effects were found for adolescent-reported parenting or adolescent depression or anxiety symptoms, although when transformed data was used, greater reduction in parent-reported adolescent depressive symptoms was observed in the intervention group (Cohen's d = -0.21; 95% CI -0.42, -0.01). Both groups showed significant reductions in anxiety (both reporters) and depressive (parent-report) symptoms."

INTRODUCTION

2a) In INTRODUCTION: Scientific background and explanation of rationale

2a-i) Problem and the type of system/solution

Describe the problem and the type of system/solution that is object of the study: intended as stand-alone intervention vs. incorporated in broader health care program? Intended for a particular patient population? Goals of the intervention, e.g., being more cost-effective to other interventions, replace or complement other solutions? (Note: Details about the intervention are provided in "Methods" under 5)

	1	2	3	4	5	
subitem not at all important	○	○	○	◉	○	essential

Does your paper address subitem 2a-i? *

Copy and paste relevant sections from the manuscript (include quotes in quotation marks "like this" to indicate direct quotes from your manuscript), or elaborate on this item by providing additional information not in the ms, or briefly explain why the item is not applicable/relevant for your study

Yes.
"The Partners in Parenting web-based parenting intervention was developed to address the abovementioned gaps in preventive parenting resources [24]. It is an evidence-informed intervention that incorporates: (1) developmental theory [including the developmental psychopathology framework; 26] and research into the role of parents in adolescent development and adjustment [10, 15], (2) preventive medicine and public health approaches that advocate the targeting of risk and protective factors for prevention [12, 27], and (3) the use of persuasive technologies to influence behavior change [28]. Partners in Parenting draws its content from the parenting guidelines How to prevent depression and clinical anxiety in your teenager: Strategies for parents [henceforce the Guidelines; 29]. These Guidelines were the product of a rigorous research translation methodology comprising a systematic review of modifiable parental factors associated with adolescent depression and anxiety [10] and a Delphi study of international expert consensus about parenting strategies that can reduce adolescents' risk of depression and anxiety disorders [30]. Using a consumer-engagement approach [31], the intervention was designed following the principles of Persuasive Systems Design [28] to be an interactive, individually-tailored program."

2a-ii) Scientific background, rationale: What is known about the (type of) system

Scientific background, rationale: What is known about the (type of) system that is the object of the study (be sure to discuss the use of similar systems for other conditions/diagnoses, if appropiate), motivation for the study, i.e. what are the reasons for and what is the context for this specific study, from which stakeholder viewpoint is the study performed, potential impact of findings [2]. Briefly justify the choice of the comparator.

	1	2	3	4	5	
subitem not at all important	○	○	◉	○	○	essential

Does your paper address subitem 2a-ii? *

Copy and paste relevant sections from the manuscript (include quotes in quotation marks "like this" to indicate direct quotes from your manuscript), or elaborate on this item by providing additional information not in the ms, or briefly explain why the item is not applicable/relevant for your study

Yes. See introduction paragraphs beginning:
Paragraph 2: "The family represents a strategic setting in which to implement preventive approaches for adolescent depression and anxiety (also known as internalizing) disorders. ..."
Paragraph 3: "For the purpose of prevention, interventions need to target modifiable risk and protective factors [12]. ..."
Paragraph 4: "For decades, preventive parenting interventions have been developed to capitalize on the influence parents have on their child's development and adjustment, based on the assumption that improving parenting will in turn yield benefits for the child's mental health [17]. ..."
Paragraph 3: "However, the public health impact of preventive parenting interventions (regardless of child age) is limited by poor uptake and engagement [21]."
Paragraph 6: "The Partners in Parenting web-based parenting intervention was developed to address the abovementioned gaps in preventive parenting resources [24]."

2b) In INTRODUCTION: Specific objectives or hypotheses

Does your paper address CONSORT subitem 2b? *

Copy and paste relevant sections from the manuscript (include quotes in quotation marks "like this" to indicate direct quotes from your manuscript), or elaborate on this item by providing additional information not in the ms, or briefly explain why the item is not applicable/relevant for your study

Yes.
"The primary outcome of interest was again parenting risk and protective factors. We hypothesized that the intervention effects observed at post-intervention would be observed at 12-month follow-up. Parent- and adolescent-reported symptoms were also examined as primary outcomes in the current paper The same set of secondary outcomes reported for post intervention was examined, with the aim of investigating whether the intervention effects on parent-reported parenting factors would translate into adolescent-reported changes in parenting, as well as yield benefits in terms of adolescent depressive and anxiety symptoms. Specifically, we hypothesized that, compared to the control group, the intervention group would show greater improvements in adolescent-reported parenting factors and greater reductions in parent- and adolescent-reported symptoms from baseline to 12-month follow-up. We also hypothesized that parenting at post-intervention would mediate adolescent symptoms at 12-month follow-up, after accounting for parenting and symptom scores at baseline. Adolescent-report of parenting was again examined as a secondary outcome measure. We predicted greater improvement in adolescent-reported parenting from baseline to 12-month follow-up in the intervention compared to control group."

METHODS

3a) Description of trial design (such as parallel, factorial) including allocation ratio

Does your paper address CONSORT subitem 3a? *

Copy and paste relevant sections from the manuscript (include quotes in quotation marks "like this" to indicate direct quotes from your manuscript), or elaborate on this item by providing additional information not in the ms, or briefly explain why the item is not applicable/relevant for your study

Yes.
"The current study was a parallel-group superiority RCT, with assessments conducted at baseline (pre-intervention), post-intervention (3-months post-baseline), and 12-month follow-up (final assessment timepoint)."

3b) Important changes to methods after trial commencement (such as eligibility criteria), with reasons

Does your paper address CONSORT subitem 3b? *

Copy and paste relevant sections from the manuscript (include quotes in quotation marks "like this" to indicate direct quotes from your manuscript), or elaborate on this item by providing additional information not in the ms, or briefly explain why the item is not applicable/relevant for your study

Not applicable, there were no changes to the methods after trial commencement.

3b-i) Bug fixes, Downtimes, Content Changes

Bug fixes, Downtimes, Content Changes: ehealth systems are often dynamic systems. A description of changes to methods therefore also includes important changes made on the intervention or comparator during the trial (e.g., major bug fixes or changes in the functionality or content) (5-iii) and other “unexpected events” that may have influenced study design such as staff changes, system failures/downtimes, etc. [2].

	1	2	3	4	5	
subitem not at all important	○	○	○	◉	○	essential

Does your paper address subitem 3b-i?

Copy and paste relevant sections from the manuscript (include quotes in quotation marks "like this" to indicate direct quotes from your manuscript), or elaborate on this item by providing additional information not in the ms, or briefly explain why the item is not applicable/relevant for your study

Not applicable, there were no bug fixes, downtimes or content changes during the trial.

4a) Eligibility criteria for participants

Does your paper address CONSORT subitem 4a? *

Copy and paste relevant sections from the manuscript (include quotes in quotation marks "like this" to indicate direct quotes from your manuscript), or elaborate on this item by providing additional information not in the ms, or briefly explain why the item is not applicable/relevant for your study

Yes.
"Eligible parents had an adolescent in the target age range (12 to 15 years at baseline), resided in Australia, had regular internet access, and an email account. Only one parent-adolescent dyad per family was eligible to participate. Computer and internet literacy were implicit eligibility criteria."
"). Parents provided consent and contact details for their adolescent, however parents were still able to participate if their adolescent chose not to. No exclusion criteria were specified."

4a-i) Computer / Internet literacy

Computer / Internet literacy is often an implicit “de facto” eligibility criterion - this should be explicitly clarified.

	1	2	3	4	5	
subitem not at all important	○	○	○	●	○	essential

Does your paper address subitem 4a-i?

Copy and paste relevant sections from the manuscript (include quotes in quotation marks "like this" to indicate direct quotes from your manuscript), or elaborate on this item by providing additional information not in the ms, or briefly explain why the item is not applicable/relevant for your study

Yes.
"Computer and internet literacy were implicit eligibility criteria."

4a-ii) Open vs. closed, web-based vs. face-to-face assessments:

Open vs. closed, web-based vs. face-to-face assessments: Mention how participants were recruited (online vs. offline), e.g., from an open access website or from a clinic, and clarify if this was a purely web-based trial, or there were face-to-face components (as part of the intervention or for assessment), i.e., to what degree got the study team to know the participant. In online-only trials, clarify if participants were quasi-anonymous and whether having multiple identities was possible or whether technical or logistical measures (e.g., cookies, email confirmation, phone calls) were used to detect/prevent these.

	1	2	3	4	5	
subitem not at all important	○	○	○	●	○	essential

Does your paper address subitem 4a-ii? *

Copy and paste relevant sections from the manuscript (include quotes in quotation marks "like this" to indicate direct quotes from your manuscript), or elaborate on this item by providing additional information not in the ms, or briefly explain why the item is not applicable/relevant for your study

Yes.
"Recruitment was primarily via secondary schools across Australia, which distributed recruitment flyers via their usual means of communication with parents (e.g., newsletters, online parent portals, email). Recruitment materials were also disseminated via online networks, social media, and mental health organizations (e.g., beyondblue and Mental Health First Aid Australia). Interested parents registered for the trial via the dedicated trial website (see Multimedia Appendix X for informed consent documentation, which was provided via the trial website). Parents provided consent and contact details for their adolescent, however parents were still able to participate if their adolescent chose not to."
"Parent participants registered themselves and their adolescent via the dedicated trial website. Email verification was required at this point. A member of the research team then phoned the adolescent to inform them of study requirements and obtain assent (if they agreed to participate). Parents were not informed of their adolescent's decision to participate and could continue in the study regardless of adolescent participation. If the adolescent declined to take part, the adolescent assessments were cancelled, so that parent participation could proceed as per protocol. Adolescents who agreed to participate were guided through completion of their online baseline assessment over the phone, with the researcher providing assistance as required. On completion of the adolescent baseline assessment (or cancellation of the assessment by a researcher), the trial website automatically generated an email invitation to the parent, inviting them to complete their baseline assessment. Parents were then required to log on to the website in their own time to complete their baseline assessment. Parent and adolescent assessments included their respective versions of the PRADAS, SCAS, and SMFQ."

4a-iii) Information giving during recruitment

Information given during recruitment. Specify how participants were briefed for recruitment and in the informed consent procedures (e.g., publish the informed consent documentation as appendix, see also item X26), as this information may have an effect on user self-selection, user expectation and may also bias results.

	1	2	3	4	5	
subitem not at all important	○	○	○	◉	○	essential

Does your paper address subitem 4a-iii?

Copy and paste relevant sections from the manuscript (include quotes in quotation marks "like this" to indicate direct quotes from your manuscript), or elaborate on this item by providing additional information not in the ms, or briefly explain why the item is not applicable/relevant for your study

Yes. The informed consent documentation will be published as an appendix.

4b) Settings and locations where the data were collected

Does your paper address CONSORT subitem 4b? *

Copy and paste relevant sections from the manuscript (include quotes in quotation marks "like this" to indicate direct quotes from your manuscript), or elaborate on this item by providing additional information not in the ms, or briefly explain why the item is not applicable/relevant for your study

Yes.
"Eligible parents had an adolescent in the target age range (12 to 15 years at baseline), resided in Australia, had regular internet access, and an email account."
"Adolescents who agreed to participate were guided through completion of their online baseline assessment over the phone, with the researcher providing assistance as required. On completion of the adolescent baseline assessment (or cancellation of the assessment by a researcher), the trial website automatically generated an email invitation to the parent, inviting them to complete their baseline assessment. Parents were then required to log on to the website in their own time to complete their baseline assessment."

4b-i) Report if outcomes were (self-)assessed through online questionnaires

Clearly report if outcomes were (self-)assessed through online questionnaires (as common in web-based trials) or otherwise.

	1	2	3	4	5	
subitem not at all important	○	○	○	◉	○	essential

Does your paper address subitem 4b-i? *

Copy and paste relevant sections from the manuscript (include quotes in quotation marks "like this" to indicate direct quotes from your manuscript), or elaborate on this item by providing additional information not in the ms, or briefly explain why the item is not applicable/relevant for your study

Yes. All assessment were self-assessed via online surveys.
""Adolescents who agreed to participate were guided through completion of their online baseline assessment over the phone, with the researcher providing assistance as required. On completion of the adolescent baseline assessment (or cancellation of the assessment by a researcher), the trial website automatically generated an email invitation to the parent, inviting them to complete their baseline assessment. Parents were then required to log on to the website in their own time to complete their baseline assessment."

4b-ii) Report how institutional affiliations are displayed

Report how institutional affiliations are displayed to potential participants [on ehealth media], as affiliations with prestigious hospitals or universities may affect volunteer rates, use, and reactions with regards to an intervention.(Not a required item – describe only if this may bias results)

	1	2	3	4	5	
subitem not at all important	○	○	◉	○	○	essential

Does your paper address subitem 4b-ii?

Copy and paste relevant sections from the manuscript (include quotes in quotation marks "like this" to indicate direct quotes from your manuscript), or elaborate on this item by providing additional information not in the ms, or briefly explain why the item is not applicable/relevant for your study

Not directly stated, however screenshots and informed consent documentation are provided as appendices. These documents show where the university logo was included.

5) The interventions for each group with sufficient details to allow replication, including how and when they were actually administered

5-i) Mention names, credential, affiliations of the developers, sponsors, and owners

Mention names, credential, affiliations of the developers, sponsors, and owners [6] (if authors/evaluators are owners or developer of the software, this needs to be declared in a “Conflict of interest” section or mentioned elsewhere in the manuscript).

	1	2	3	4	5	
subitem not at all important	○	○	○	●	○	essential

Does your paper address subitem 5-i?

Copy and paste relevant sections from the manuscript (include quotes in quotation marks "like this" to indicate direct quotes from your manuscript), or elaborate on this item by providing additional information not in the ms, or briefly explain why the item is not applicable/relevant for your study

Yes. Developers of the intervention are included in the conflicts of interest section.
"MBHY, MCB, AFJ and KL are co-developers of the PiP intervention, and MBHY and AFJ are cofounders of the broader Parenting Strategies online platform of parenting interventions, including PiP. None of the authors derive any personal financial benefit from these interventions."

5-ii) Describe the history/development process

Describe the history/development process of the application and previous formative evaluations (e.g., focus groups, usability testing), as these will have an impact on adoption/use rates and help with interpreting results.

	1	2	3	4	5	
subitem not at all important	○	○	○	●	○	essential

Does your paper address subitem 5-ii?

Copy and paste relevant sections from the manuscript (include quotes in quotation marks "like this" to indicate direct quotes from your manuscript), or elaborate on this item by providing additional information not in the ms, or briefly explain why the item is not applicable/relevant for your study

Yes.
"The Partners in Parenting web-based parenting intervention was developed to address the abovementioned gaps in preventive parenting resources [24]. It is an evidence-informed intervention that incorporates: (1) developmental theory [including the developmental psychopathology framework; 26] and research into the role of parents in adolescent development and adjustment [10, 15], (2) preventive medicine and public health approaches that advocate the targeting of risk and protective factors for prevention [12, 27], and (3) the use of persuasive technologies to influence behavior change [28]. Partners in Parenting draws its content from the parenting guidelines How to prevent depression and clinical anxiety in your teenager: Strategies for parents [henceforce the Guidelines; 29]. These Guidelines were the product of a rigorous research translation methodology comprising a systematic review of modifiable parental factors associated with adolescent depression and anxiety [10] and a Delphi study of international expert consensus about parenting strategies that can reduce adolescents' risk of depression and anxiety disorders [30]. Using a consumer-engagement approach [31], the intervention was designed following the principles of Persuasive Systems Design [28] to be an interactive, individually-tailored program. The intervention has been evaluated in a randomized controlled trial (RCT) and found to produce significantly greater improvements in parent-reported parenting risk and protective factors for adolescent depression and anxiety (primary outcome) from baseline to post-intervention (3 months later), compared to an active-control condition (Cohen's d = 0.57). No significant group differences in changes over time were found for secondary outcomes of interest, including adolescent-reported parenting factors, and adolescent depression and anxiety symptoms as reported by both parent and adolescent [32]. It is likely that changes on these secondary outcomes are more likely to emerge in the longer term, once the behavior changes in parents have had time to influence the broader family system."

5-iii) Revisions and updating

Revisions and updating. Clearly mention the date and/or version number of the application/intervention (and comparator, if applicable) evaluated, or describe whether the intervention underwent major changes during the evaluation process, or whether the development and/or content was "frozen" during the trial. Describe dynamic components such as news feeds or changing content which may have an impact on the replicability of the intervention (for unexpected events see item 3b).

	1	2	3	4	5	
subitem not at all important	○	○	◉	○	○	essential

Does your paper address subitem 5-iii?

Copy and paste relevant sections from the manuscript (include quotes in quotation marks "like this" to indicate direct quotes from your manuscript), or elaborate on this item by providing additional information not in the ms, or briefly explain why the item is not applicable/relevant for your study

Not applicable. There were no revisions or updating of the intervention.

5-iv) Quality assurance methods

Provide information on quality assurance methods to ensure accuracy and quality of information provided [1], if applicable.

	1	2	3	4	5	
subitem not at all important	○	○	◉	○	○	essential

Does your paper address subitem 5-iv?

Copy and paste relevant sections from the manuscript (include quotes in quotation marks "like this" to indicate direct quotes from your manuscript), or elaborate on this item by providing additional information not in the ms, or briefly explain why the item is not applicable/relevant for your study

Not applicable.

5-v) Ensure replicability by publishing the source code, and/or providing screenshots/screen-capture video, and/or providing flowcharts of the algorithms used

Ensure replicability by publishing the source code, and/or providing screenshots/screen-capture video, and/or providing flowcharts of the algorithms used. Replicability (i.e., other researchers should in principle be able to replicate the study) is a hallmark of scientific reporting.

	1	2	3	4	5	
subitem not at all important	○	○	◉	○	○	essential

Does your paper address subitem 5-v?

Copy and paste relevant sections from the manuscript (include quotes in quotation marks "like this" to indicate direct quotes from your manuscript), or elaborate on this item by providing additional information not in the ms, or briefly explain why the item is not applicable/relevant for your study

Source code/algorithms not provided due to IP.
Screenshots of the intervention will be provided as an appendix.

5-vi) Digital preservation

Digital preservation: Provide the URL of the application, but as the intervention is likely to change or disappear over the course of the years; also make sure the intervention is archived (Internet Archive, webcitation.org, and/or publishing the source code or screenshots/videos alongside the article). As pages behind login screens cannot be archived, consider creating demo pages which are accessible without login.

	1	2	3	4	5	
subitem not at all important	○	○	◉	○	○	essential

Does your paper address subitem 5-vi?

Copy and paste relevant sections from the manuscript (include quotes in quotation marks "like this" to indicate direct quotes from your manuscript), or elaborate on this item by providing additional information not in the ms, or briefly explain why the item is not applicable/relevant for your study

Not applicable.
The intervention URL is no longer active, and can no longer be accessed publicly.
Screenshots of the intervention will be published as appendices.

5-vii) Access

Access: Describe how participants accessed the application, in what setting/context, if they had to pay (or were paid) or not, whether they had to be a member of specific group. If known, describe how participants obtained “access to the platform and Internet” [1]. To ensure access for editors/reviewers/readers, consider to provide a “backdoor” login account or demo mode for reviewers/readers to explore the application (also important for archiving purposes, see vi).

	1	2	3	4	5	
subitem not at all important	○	○	◉	○	○	essential

Does your paper address subitem 5-vii? *

Copy and paste relevant sections from the manuscript (include quotes in quotation marks "like this" to indicate direct quotes from your manuscript), or elaborate on this item by providing additional information not in the ms, or briefly explain why the item is not applicable/relevant for your study

Yes.
Intervention access: "The Partners in Parenting Intervention (PiP). PiP is a fully-automated, web-based parenting program consisting of three components [24]. First, parents complete a self-assessment scale (the Parenting to Reduce Adolescent Depression and Anxiety Scale [PRADAS] [33]), which assesses their current parenting practices against the Guidelines. Second, based on their responses to the PRADAS, parents receive an individually-tailored feedback report outlining parenting strengths and areas for improvement. The feedback messages are designed to be brief, motivate behavior change, provide practical parenting strategies, and contain links to further information that parents can access if desired. Third, parents are recommended a series of interactive online modules, also based on their responses to the PRADAS. A total of nine modules are available, and parents can further personalize their program by selecting additional modules (not initially recommended to them) or declining recommended modules. Modules include interactive activities, goal setting exercises, audio clips, vignettes, illustrations, and an end-of-module quiz with immediate feedback, designed to consolidate learning. Each module takes approximately 15-25 minutes to complete, depending on how the parent chooses to engage. One module is made available to parents every 7 days, to allow sufficient time to complete each module and work on weekly goals before progressing to the next module. Parents were sent automated emails each week to notify them that their next module was available to access via their personalized dashboard. Once parents had completed the initially selected modules, all nine modules (including those not initially selected) were made available for the remainder of the RCT. Table 1 presents the Guidelines' topics, corresponding sections of the PRADAS, title of modules, and an outline of content covered. Multimedia Appendix 1 presents screenshots of the intervention."
Educational factsheets (active control condition). Parents in the control group received access to a series of five educational factsheets about adolescent development and mental health. The factsheets provided general information, without individual tailoring or actionable parenting strategies (cf. the PiP intervention). The factsheets were intended to provide information already available to parents as part of a current health promotion approach, with materials adapted from the Raising Children Network website [34]. The factsheet topics were: 1) Teen development: an overview; 2) The teenager's developing brain; 3) The teenager's changing body; 4) Resilience; and 5) Happy teenagers and teenage wellbeing. The delivery of the factsheets was designed to mirror the

delivery of the PiP intervention; parents were emailed once per week with a link to access their next factsheet via their personal dashboard on the trial website, and had access to the factsheets for the duration of the RCT."
Payment: "Both parents and adolescents were reimbursed with an AUD$15 e-voucher for completion of each of the 3 and 12-month follow-up assessments."

5-viii) Mode of delivery, features/functionalities/components of the intervention and comparator, and the theoretical framework

Describe mode of delivery, features/functionalities/components of the intervention and comparator, and the theoretical framework [6] used to design them (instructional strategy [1], behaviour change techniques, persuasive features, etc., see e.g., [7, 8] for terminology). This includes an in-depth description of the content (including where it is coming from and who developed it) [1]," whether [and how] it is tailored to individual circumstances and allows users to track their progress and receive feedback" [6]. This also includes a description of communication delivery channels and – if computer-mediated communication is a component – whether communication was synchronous or asynchronous [6]. It also includes information on presentation strategies [1], including page design principles, average amount of text on pages, presence of hyperlinks to other resources, etc. [1].

	1	2	3	4	5	
subitem not at all important	○	○	○	●	○	essential

Does your paper address subitem 5-viii? *

Copy and paste relevant sections from the manuscript (include quotes in quotation marks "like this" to indicate direct quotes from your manuscript), or elaborate on this item by providing additional information not in the ms, or briefly explain why the item is not applicable/relevant for your study

Yes.
From the Introduction section:
"The Partners in Parenting web-based parenting intervention was developed to address the abovementioned gaps in preventive parenting resources [24]. It is an evidence-informed intervention that incorporates: (1) developmental theory [including the developmental psychopathology framework; 26] and research into the role of parents in adolescent development and adjustment [10, 15], (2) preventive medicine and public health approaches that advocate the targeting of risk and protective factors for prevention [12, 27], and (3) the use of persuasive technologies to influence behavior change [28]. Partners in Parenting draws its content from the parenting guidelines How to prevent depression and clinical anxiety in your teenager: Strategies for parents [henceforce the Guidelines; 29]. These Guidelines were the product of a rigorous research translation methodology comprising a systematic review of modifiable parental factors associated with adolescent depression and anxiety [10] and a Delphi study of international expert consensus about parenting strategies that can reduce adolescents' risk of depression and anxiety disorders [30]. Using a consumer-engagement approach [31], the intervention was designed following the principles of Persuasive Systems Design [28] to be an interactive, individually-tailored program."
From the Methods section:
"The Partners in Parenting Intervention (PiP). PiP is a fully-automated, web-based parenting program consisting of three components [24]. First, parents complete a self-assessment scale (the Parenting to Reduce Adolescent Depression and Anxiety Scale [PRADAS] [33]), which assesses their current parenting practices against the Guidelines. Second, based on their responses to the PRADAS, parents receive an individually-tailored feedback report outlining parenting strengths and areas for improvement. The feedback messages are designed to be brief, motivate behavior change, provide practical parenting strategies, and contain links to further information that parents can access if desired. Third, parents are recommended a series of interactive online modules, also based on their responses to the PRADAS. A total of nine modules are available, and parents can further personalize their program by selecting additional modules (not initially recommended to them) or declining recommended modules. Modules include interactive activities, goal setting exercises, audio clips, vignettes, illustrations, and an end-of-module quiz with immediate feedback, designed to consolidate learning. Each module takes approximately 15-25 minutes to complete, depending on how the parent chooses to engage. One module is made available to parents every 7 days, to allow sufficient time to complete each module and

work on weekly goals before progressing to the next module. Parents were sent automated emails each week to notify them that their next module was available to access via their personalized dashboard. Once parents had completed the initially selected modules, all nine modules (including those not initially selected) were made available for the remainder of the RCT. Table 1 presents the Guidelines' topics, corresponding sections of the PRADAS, title of modules, and an outline of content covered. Multimedia Appendix 1 presents screenshots of the intervention."

5-ix) Describe use parameters

Describe use parameters (e.g., intended "doses" and optimal timing for use). Clarify what instructions or recommendations were given to the user, e.g., regarding timing, frequency, heaviness of use, if any, or was the intervention used ad libitum.

	1	2	3	4	5	
subitem not at all important	○	○	○	◉	○	essential

Does your paper address subitem 5-ix?

Copy and paste relevant sections from the manuscript (include quotes in quotation marks "like this" to indicate direct quotes from your manuscript), or elaborate on this item by providing additional information not in the ms, or briefly explain why the item is not applicable/relevant for your study

Yes.
"Each module takes approximately 15-25 minutes to complete, depending on how the parent chooses to engage. One module is made available to parents every 7 days, to allow sufficient time to complete each module and work on weekly goals before progressing to the next module. Once parents had completed the initially selected modules, all nine modules (including those not initially selected) were made available for the remainder of the RCT."

5-x) Clarify the level of human involvement

Clarify the level of human involvement (care providers or health professionals, also technical assistance) in the e-intervention or as co-intervention (detail number and expertise of professionals involved, if any, as well as "type of assistance offered, the timing and frequency of the support, how it is initiated, and the medium by which the assistance is delivered". It may be necessary to distinguish between the level of human involvement required for the trial, and the level of human involvement required for a routine application outside of a RCT setting (discuss under item 21 – generalizability).

	1	2	3	4	5	
subitem not at all important	○	○	○	◉	○	essential

Does your paper address subitem 5-x?

Copy and paste relevant sections from the manuscript (include quotes in quotation marks "like this" to indicate direct quotes from your manuscript), or elaborate on this item by providing additional information not in the ms, or briefly explain why the item is not applicable/relevant for your study

Yes.
"Weekly check-in phone calls
Parents in both groups received a weekly phone call from a member of the research team, commencing 7 days after completion of the baseline assessment. Intervention group parents received one phone call per module selected in their program, unless they selected less than five modules, in which case parents received a minimum of five calls (to match the control group). Control group parents received five calls, to align with the number of factsheets. The purpose of the calls was to encourage progress through the program, enhance engagement, provide technical assistance as required, and answer study-related questions. Researchers were trained to make the phone calls following a standard script, and did not provide individual advice or therapeutic support."

5-xi) Report any prompts/reminders used

Report any prompts/reminders used: Clarify if there were prompts (letters, emails, phone calls, SMS) to use the application, what triggered them, frequency etc. It may be necessary to distinguish between the level of prompts/reminders required for the trial, and the level of prompts/reminders for a routine application outside of a RCT setting (discuss under item 21 – generalizability).

	1	2	3	4	5	
subitem not at all important	○	○	○	●	○	essential

Does your paper address subitem 5-xi? *

Copy and paste relevant sections from the manuscript (include quotes in quotation marks "like this" to indicate direct quotes from your manuscript), or elaborate on this item by providing additional information not in the ms, or briefly explain why the item is not applicable/relevant for your study

Yes.
Intervention reminders: "Parents were sent automated emails each week to notify them that their next module was available to access via their personalized dashboard."
"The delivery of the factsheets was designed to mirror the delivery of the PiP intervention; parents were emailed once per week with a link to access their next factsheet via their personal dashboard on the trial website, and had access to the factsheets for the duration of the RCT."
Assessment reminders: "...the trial website automatically generated an email invitation to the parent, inviting them to complete their baseline assessment. Parents were then required to log on to the website in their own time to complete their baseline assessment."

5-xii) Describe any co-interventions (incl. training/support)

Describe any co-interventions (incl. training/support): Clearly state any interventions that are provided in addition to the targeted eHealth intervention, as ehealth intervention may not be designed as stand-alone intervention. This includes training sessions and support [1]. It may be necessary to distinguish between the level of training required for the trial, and the level of training for a routine application outside of a RCT setting (discuss under item 21 – generalizability.

	1	2	3	4	5	
subitem not at all important	○	○	●	○	○	essential

Does your paper address subitem 5-xii? *

Copy and paste relevant sections from the manuscript (include quotes in quotation marks "like this" to indicate direct quotes from your manuscript), or elaborate on this item by providing additional information not in the ms, or briefly explain why the item is not applicable/relevant for your study

Not applicable, there were no co-interventions.

6a) Completely defined pre-specified primary and secondary outcome measures, including how and when they were assessed

Does your paper address CONSORT subitem 6a? *

Copy and paste relevant sections from the manuscript (include quotes in quotation marks "like this" to indicate direct quotes from your manuscript), or elaborate on this item by providing additional information not in the ms, or briefly explain why the item is not applicable/relevant for your study

Yes.
"The primary outcome of interest was again parenting risk and protective factors. We hypothesized that the intervention effects observed at post-intervention would be observed at 12-month follow-up. Parent- and adolescent-reported symptoms were also examined as primary outcomes in the current paper The same set of secondary outcomes reported for post intervention was examined, with the aim of investigating whether the intervention effects on parent-reported parenting factors would translate into adolescent-reported changes in parenting, as well as yield benefits in terms of adolescent depressive and anxiety symptoms. Specifically, we hypothesized that, compared to the control group, the intervention group would show greater improvements in adolescent-reported parenting factors and greater reductions in parent- and adolescent-reported symptoms from baseline to 12-month follow-up. We also hypothesized that parenting at post-intervention would mediate adolescent symptoms at 12-month follow-up, after accounting for parenting and symptom scores at baseline. Adolescent-report of parenting was again examined as a secondary outcome measure. We predicted greater improvement in adolescent-reported parenting from baseline to 12-month follow-up in the intervention compared to control group."
"The current study was a parallel-group superiority RCT, with assessments conducted at baseline (pre-intervention), post-intervention (3-months post-baseline), and 12-month follow-up (final assessment timepoint)."
"The procedure for 3 and 12-month follow-up assessments was similar to the baseline procedure."
"Parent and adolescent assessments included their respective versions of the PRADAS, SCAS, and SMFQ."

6a-i) Online questionnaires: describe if they were validated for online use and apply CHERRIES items to describe how the questionnaires were designed/deployed

If outcomes were obtained through online questionnaires, describe if they were validated for online use and apply CHERRIES items to describe how the questionnaires were designed/deployed [9].

	1	2	3	4	5	
subitem not at all important	○	○	●	○	○	essential

Does your paper address subitem 6a-i?

Copy and paste relevant sections from manuscript text

We have followed the CHERRIES checklist as far as is applicable to our study.

6a-ii) Describe whether and how “use” (including intensity of use/dosage) was defined/measured/monitored

Describe whether and how “use” (including intensity of use/dosage) was defined/measured/monitored (logins, logfile analysis, etc.). Use/adoption metrics are important process outcomes that should be reported in any ehealth trial.

	1	2	3	4	5	
subitem not at all important	○	○	○	◉	○	essential

Does your paper address subitem 6a-ii?

Copy and paste relevant sections from manuscript text

Yes.
"Intervention adherence, completion, and access during follow-up period. Intervention adherence was operationalized as the percentage of parents who completed their program as intended, calculated as: 100% × [(number of parents whose observed usage equals their intended usage) / (number of parents who received the intervention)] [32, 43]. Intervention completion was defined as the percentage of intended program that was completed; that is: intervention completion = [100% × (observed usage) / (intended usage)]. For the intervention group, observed usage was defined as the number of modules completed, and intended usage as the number of modules initially selected. Module completion was determined based on one of three criteria being met: 1) a "close timestamp" was stored, indicating that the parent clicked the "finish module" button on the last page of the module; 2) responses were entered for the end-of-module quiz; or 3) the goal selected for the module was marked as completed by the parent. For the control group, intended usage was defined as reading all five factsheets, and observed usage was defined as the number of factsheets that had been opened by the parent (determined by timestamps stored in the system when parents clicked the link to open their factsheet). We also examined whether parents completed their program during the active intervention phase, which was defined as the time between the parent baseline assessment and the adolescent post-intervention. If the adolescent did not complete the assessment, the date of the parent post-intervention assessment was used. Finally, we examined the number of parents who accessed their program after the active intervention phase (i.e., between post-intervention and 12-month assessment time points)."

6a-iii) Describe whether, how, and when qualitative feedback from participants was obtained

Describe whether, how, and when qualitative feedback from participants was obtained (e.g., through emails, feedback forms, interviews, focus groups).

	1	2	3	4	5	
subitem not at all important	○	○	◉	○	○	essential

Does your paper address subitem 6a-iii?

Copy and paste relevant sections from manuscript text

Not applicable, we did not obtain qualitative feedback.

6b) Any changes to trial outcomes after the trial commenced, with reasons

Does your paper address CONSORT subitem 6b? *

Copy and paste relevant sections from the manuscript (include quotes in quotation marks "like this" to indicate direct quotes from your manuscript), or elaborate on this item by providing additional information not in the ms, or briefly explain why the item is not applicable/relevant for your study

Not applicable, there were no changes to trial outcomes after the trial commenced.

7a) How sample size was determined

NPT: When applicable, details of whether and how the clustering by care provides or centers was addressed

7a-i) Describe whether and how expected attrition was taken into account when calculating the sample size

Describe whether and how expected attrition was taken into account when calculating the sample size.

	1	2	3	4	5	
subitem not at all important	○	○	○	◉	○	essential

Does your paper address subitem 7a-i?

Copy and paste relevant sections from manuscript title (include quotes in quotation marks "like this" to indicate direct quotes from your manuscript), or elaborate on this item by providing additional information not in the ms, or briefly explain why the item is not applicable/relevant for your study

Yes.
"Sample size was determined based on an a priori power analysis. This indicated that a sample size of 294 parent-adolescent dyads (147 per group) was required to detect a small effect size (Cohen's d = 0.20), with an alpha level of .05, power of 0.80, and a repeated measures design. To allow for approximately 15% attrition, we aimed to recruit 338 dyads (169 per group)."

7b) When applicable, explanation of any interim analyses and stopping guidelines

Does your paper address CONSORT subitem 7b? *

Copy and paste relevant sections from the manuscript (include quotes in quotation marks "like this" to indicate direct quotes from your manuscript), or elaborate on this item by providing additional information not in the ms, or briefly explain why the item is not applicable/relevant for your study

Not applicable, there were no interim analyses or stopping guidelines.
Recruitment ended when the desired sample size was reached.

8a) Method used to generate the random allocation sequence

NPT: When applicable, how care providers were allocated to each trial group

Does your paper address CONSORT subitem 8a? *

Copy and paste relevant sections from the manuscript (include quotes in quotation marks "like this" to indicate direct quotes from your manuscript), or elaborate on this item by providing additional information not in the ms, or briefly explain why the item is not applicable/relevant for your study

Yes.
"Randomization and blinding. Immediately following completion of the parent baseline assessment, parents were automatically allocated to the intervention or control condition using a computer generated unblocked, unstratified randomization procedure, with a 1:1 allocation ratio."

8b) Type of randomisation; details of any restriction (such as blocking and block size)

Does your paper address CONSORT subitem 8b? *

Copy and paste relevant sections from the manuscript (include quotes in quotation marks "like this" to indicate direct quotes from your manuscript), or elaborate on this item by providing additional information not in the ms, or briefly explain why the item is not applicable/relevant for your study

Yes.
"Randomization and blinding. Immediately following completion of the parent baseline assessment, parents were automatically allocated to the intervention or control condition using a computer generated unblocked, unstratified randomization procedure, with a 1:1 allocation ratio."

9) Mechanism used to implement the random allocation sequence (such as sequentially numbered containers), describing any steps taken to conceal the sequence until interventions were assigned

Does your paper address CONSORT subitem 9? *

Copy and paste relevant sections from the manuscript (include quotes in quotation marks "like this" to indicate direct quotes from your manuscript), or elaborate on this item by providing additional information not in the ms, or briefly explain why the item is not applicable/relevant for your study

Yes.
"Randomization and blinding. Immediately following completion of the parent baseline assessment, parents were automatically allocated to the intervention or control condition using a computer generated unblocked, unstratified randomization procedure, with a 1:1 allocation ratio. At this point, intervention group parents were presented with their individually-tailored feedback on screen, and were emailed a pdf copy of the feedback report. Control group parents were presented with their first factsheet. Parents were therefore not blinded to their allocation, nor were the researchers who spoke to parents during weekly check-in phone calls. Adolescents were not informed of their parent's allocation, therefore were assumed to be blinded. As all assessments were completed online via the dedicated trial website, blinding of assessor was not relevant."

10) Who generated the random allocation sequence, who enrolled participants, and who assigned participants to interventions

Does your paper address CONSORT subitem 10? *

Copy and paste relevant sections from the manuscript (include quotes in quotation marks "like this" to indicate direct quotes from your manuscript), or elaborate on this item by providing additional information not in the ms, or briefly explain why the item is not applicable/relevant for your study

Yes.
"Randomization and blinding. Immediately following completion of the parent baseline assessment, parents were automatically allocated to the intervention or control condition using a computer generated unblocked, unstratified randomization procedure, with a 1:1 allocation ratio. At this point, intervention group parents were presented with their individually-tailored feedback on screen, and were emailed a pdf copy of the feedback report. Control group parents were presented with their first factsheet. Parents were therefore not blinded to their allocation, nor were the researchers who spoke to parents during weekly check-in phone calls. Adolescents were not informed of their parent's allocation, therefore were assumed to be blinded. As all assessments were completed online via the dedicated trial website, blinding of assessor was not relevant."

11a) If done, who was blinded after assignment to interventions (for example, participants, care providers, those assessing outcomes) and how

NPT: Whether or not administering co-interventions were blinded to group assignment

11a-i) Specify who was blinded, and who wasn't

Specify who was blinded, and who wasn't. Usually, in web-based trials it is not possible to blind the participants [1, 3] (this should be clearly acknowledged), but it may be possible to blind outcome assessors, those doing data analysis or those administering co-interventions (if any).

	1	2	3	4	5	
subitem not at all important	○	○	○	○	◉	essential

Does your paper address subitem 11a-i? *

Copy and paste relevant sections from the manuscript (include quotes in quotation marks "like this" to indicate direct quotes from your manuscript), or elaborate on this item by providing additional information not in the ms, or briefly explain why the item is not applicable/relevant for your study

Yes.
"Parents were therefore not blinded to their allocation, nor were the researchers who spoke to parents during weekly check-in phone calls. Adolescents were not informed of their parent’s allocation, therefore were assumed to be blinded. As all assessments were completed online via the dedicated trial website, blinding of assessor was not relevant."

11a-ii) Discuss e.g., whether participants knew which intervention was the “intervention of interest” and which one was the “comparator”

Informed consent procedures (4a-ii) can create biases and certain expectations - discuss e.g., whether participants knew which intervention was the “intervention of interest” and which one was the “comparator”.

	1	2	3	4	5	
subitem not at all important	○	○	○	◉	○	essential

Does your paper address subitem 11a-ii?

Copy and paste relevant sections from the manuscript (include quotes in quotation marks "like this" to indicate direct quotes from your manuscript), or elaborate on this item by providing additional information not in the ms, or briefly explain why the item is not applicable/relevant for your study

Yes.
"Parents were therefore not blinded to their allocation, nor were the researchers who spoke to parents during weekly check-in phone calls."

11b) If relevant, description of the similarity of interventions

(this item is usually not relevant for ehealth trials as it refers to similarity of a placebo or sham intervention to a active medication/intervention)

Does your paper address CONSORT subitem 11b? *

Copy and paste relevant sections from the manuscript (include quotes in quotation marks "like this" to indicate direct quotes from your manuscript), or elaborate on this item by providing additional information not in the ms, or briefly explain why the item is not applicable/relevant for your study

Not applicable, the interventions were not designed to be similar.

12a) Statistical methods used to compare groups for primary and secondary outcomes

NPT: When applicable, details of whether and how the clustering by care providers or centers was addressed

Does your paper address CONSORT subitem 12a? *

Copy and paste relevant sections from the manuscript (include quotes in quotation marks "like this" to indicate direct quotes from your manuscript), or elaborate on this item by providing additional information not in the ms, or briefly explain why the item is not applicable/relevant for your study

Yes.
"Primary and secondary outcome analyses were conducted on an intention-to-treat basis, using Mixed Model Repeated Measures (MMRM), with an unstructured covariance matrix. MMRM uses all available data from all participants, including those who withdrew from follow-up assessments [46]. It is a preferred analytic approach for repeated measures designs when data are considered missing at random or missing completely at random [46, 47]. As our hypotheses related to change from baseline to 12-month follow-up, we specified planned contrast tests of the group × measurement-occasion interaction from baseline to 12-month follow-up, within the overall group × measurement-occasion mixed model. This was the primary result of interest. Pairwise comparisons between groups at trial endpoint (12-month follow-up) were also examined. Cohen's d effect sizes with 95% confidence intervals (CIs) are reported for all analyses."

12a-i) Imputation techniques to deal with attrition / missing values

Imputation techniques to deal with attrition / missing values: Not all participants will use the intervention/comparator as intended and attrition is typically high in ehealth trials. Specify how participants who did not use the application or dropped out from the trial were treated in the statistical analysis (a complete case analysis is strongly discouraged, and simple imputation techniques such as LOCF may also be problematic [4]).

	1	2	3	4	5	
subitem not at all important	○	○	○	○	●	essential

Does your paper address subitem 12a-i? *

Copy and paste relevant sections from the manuscript (include quotes in quotation marks "like this" to indicate direct quotes from your manuscript), or elaborate on this item by providing additional information not in the ms, or briefly explain why the item is not applicable/relevant for your study

Yes.
"Less than 4% of participants had missing data on any measures. Item level missing data were replaced with the participant’s mean response on the corresponding subscale for cases with less than 23% missing data on a given measure. This is considered an appropriate method of imputation for this amount of missing data [45]. For cases with greater than 23% of missing items on a measure, the measure was considered missing entirely and excluded from analyses."
"Primary and secondary outcome analyses were conducted on an intention-to-treat basis, using Mixed Model Repeated Measures (MMRM), with an unstructured covariance matrix. MMRM uses all available data from all participants, including those who withdrew from follow-up assessments [46]. It is a preferred analytic approach for repeated measures designs when data are considered missing at random or missing completely at random [46, 47]."

12b) Methods for additional analyses, such as subgroup analyses and adjusted analyses

Does your paper address CONSORT subitem 12b? *

Copy and paste relevant sections from the manuscript (include quotes in quotation marks "like this" to indicate direct quotes from your manuscript), or elaborate on this item by providing additional information not in the ms, or briefly explain why the item is not applicable/relevant for your study

Yes.
"To assess for potential mediation of intervention effects on adolescent symptoms by change in parenting, we conducted simple mediation analyses using the PROCESS macro for SPSS [48, 49]. Separate mediation models were run for each symptom measure (i.e., outcome variable), with 5000 bootstrap samples for bias-corrected bootstrap 95% confidence intervals. In each model, group (coded as 0 = control, 1 = intervention) was entered as the predictor variable, 12-month symptom measure score as the outcome variable, and post-intervention PRADAS score as the mediator variable. Baseline PRADAS and baseline symptom measure score (corresponding to the outcome variable) were entered as covariates.
Finally, we conducted post hoc moderation analyses to explore moderation of intervention effects of adolescent age, gender, and baseline symptoms on outcomes. For age and baseline symptoms, the continuous moderator variable (i.e., child age at registration or baseline symptom score) was added to the mixed model as a covariate, including a three-way interaction term (i.e., group × measurement-occasion × moderator) whose significance constituted a test of a differential effect of the moderator on outcome of the intervention. Significant moderation effects were interpreted using estimated marginal means plotted for values of the covariate (i.e., moderator variable) at its 25th and 75th percentile at baseline in the sample. In order to minimize shared method variance effects, for moderation analyses using baseline symptom measures we used the symptom measure reported by the opposite informant as the moderator variable, (e.g., for the outcome of SMFQ-P [parent], baseline SMFQ-C [child] score was used as the moderator). For the parenting measures, we conducted two moderation analyses, with each of the symptom measures (SCAS and SMFQ, opposite informant to outcome measure) entered in separate models. Moderation by child gender was assessed in a similar manner using gender as an additional factor rather than as a covariate."

X26) REB/IRB Approval and Ethical Considerations [recommended as subheading under "Methods"] (not a CONSORT item)

X26-i) Comment on ethics committee approval

	1	2	3	4	5	
subitem not at all important	○	○	○	◉	○	essential

Does your paper address subitem X26-i?

Copy and paste relevant sections from the manuscript (include quotes in quotation marks "like this" to indicate direct quotes from your manuscript), or elaborate on this item by providing additional information not in the ms, or briefly explain why the item is not applicable/relevant for your study

Yes.
"The trial was prospectively registered with the Australian New Zealand Clinical Trials Registry (registration number ANZCTR12615000328572) and approved by the Monash University Human Research Ethics Committee (CF14/3887-2014002024)."

x26-ii) Outline informed consent procedures

Outline informed consent procedures e.g., if consent was obtained offline or online (how? Checkbox, etc.?), and what information was provided (see 4a-ii). See [6] for some items to be included in informed consent documents.

	1	2	3	4	5	
subitem not at all important	○	○	○	◉	○	essential

Does your paper address subitem X26-ii?

Copy and paste relevant sections from the manuscript (include quotes in quotation marks "like this" to indicate direct quotes from your manuscript), or elaborate on this item by providing additional information not in the ms, or briefly explain why the item is not applicable/relevant for your study

Yes.
"Interested parents registered for the trial via the dedicated trial website (see Multimedia Appendix X for informed consent documentation, which was provided via the trial website). Parents provided consent and contact details for their adolescent, however parents were still able to participate if their adolescent chose not to."
The informed consent documentation will be published as an appendix.

X26-iii) Safety and security procedures

Safety and security procedures, incl. privacy considerations, and any steps taken to reduce the likelihood or detection of harm (e.g., education and training, availability of a hotline)

	1	2	3	4	5	
subitem not at all important	○	○	○	◉	○	essential

Does your paper address subitem X26-iii?

Copy and paste relevant sections from the manuscript (include quotes in quotation marks "like this" to indicate direct quotes from your manuscript), or elaborate on this item by providing additional information not in the ms, or briefly explain why the item is not applicable/relevant for your study

Yes.
"At all time points, participants were followed up by a member of the research team if both the parent and adolescent reported elevated symptoms on the SCAS or SMFQ, based on pre-determined cut-off scores. For the SCAS, this was defined as a total score ≥ 1.5 SDs above the mean based on Australian community sample norms [44]. For the SMFQ, scores ≥ 8 were considered elevated [35]. Follow-up actions included email notifications to parents alerting them to their adolescent's elevated symptoms and providing avenues for seeking professional support. Adolescents who reported particularly high scores on the SMFQ (SMFQ-C total score >20) were also phoned by a postgraduate clinical psychology student, who conducted a risk assessment and provided referral information as required."

RESULTS

13a) For each group, the numbers of participants who were randomly assigned, received intended treatment, and were analysed for the primary outcome

NPT: The number of care providers or centers performing the intervention in each group and the number of patients treated by each care provider in each center

Does your paper address CONSORT subitem 13a? *

Copy and paste relevant sections from the manuscript (include quotes in quotation marks "like this" to indicate direct quotes from your manuscript), or elaborate on this item by providing additional information not in the ms, or briefly explain why the item is not applicable/relevant for your study

Yes.
"As shown in Figure 1, of the 359 parent participants who completed baseline assessments and were randomized, 319 completed post-intervention assessments, and 317 went on to complete 12-month follow-up (intervention group n = 158, control group n = 159). The number of parents reported to complete post-intervention differs from the original RCT outcome paper (previously reported as n = 318), due to an error detected when preparing the 12-month data. A parent in the intervention group was excluded from the original paper due to missing individual item response data (missing 9 items [12.3%] of the post-intervention PRADAS), however in the present paper the missing items were imputed, allowing this participant to be included in the analyses. For adolescent participants, 332 completed baseline assessments, 308 completed post-intervention assessments, and 287 completed 12-month follow-up. The attrition rate at 12-month follow-up was therefore 11.7% for parents and 13.6% for adolescents. This did not differ between conditions for parents (11.7% in each group) or adolescents (intervention group: 13.5%; control group: 13.6%). Figure 1 presents the participant flow diagram."

13b) For each group, losses and exclusions after randomisation, together with reasons

Does your paper address CONSORT subitem 13b? (NOTE: Preferably, this is shown in a CONSORT flow diagram) *

Copy and paste relevant sections from the manuscript (include quotes in quotation marks "like this" to indicate direct quotes from your manuscript), or elaborate on this item by providing additional information not in the ms, or briefly explain why the item is not applicable/relevant for your study

Yes.
"As shown in Figure 1, of the 359 parent participants who completed baseline assessments and were randomized, 319 completed post-intervention assessments, and 317 went on to complete 12-month follow-up (intervention group n = 158, control group n = 159). The number of parents reported to complete post-intervention differs from the original RCT outcome paper (previously reported as n = 318), due to an error detected when preparing the 12-month data. A parent in the intervention group was excluded from the original paper due to missing individual item response data (missing 9 items [12.3%] of the post-intervention PRADAS), however in the present paper the missing items were imputed, allowing this participant to be included in the analyses. For adolescent participants, 332 completed baseline assessments, 308 completed post-intervention assessments, and 287 completed 12-month follow-up. The attrition rate at 12-month follow-up was therefore 11.7% for parents and 13.6% for adolescents. This did not differ between conditions for parents (11.7% in each group) or adolescents (intervention group: 13.5%; control group: 13.6%). Figure 1 presents the participant flow diagram."

13b-i) Attrition diagram

Strongly recommended: An attrition diagram (e.g., proportion of participants still logging in or using the intervention/comparator in each group plotted over time, similar to a survival curve) or other figures or tables demonstrating usage/dose/engagement.

	1	2	3	4	5	
subitem not at all important	○	○	○	○	◉	essential

Does your paper address subitem 13b-i?

Copy and paste relevant sections from the manuscript or cite the figure number if applicable (include quotes in quotation marks "like this" to indicate direct quotes from your manuscript), or elaborate on this item by providing additional information not in the ms, or briefly explain why the item is not applicable/relevant for your study

Yes. A CONSORT participant flow diagram is provided, see Figure 1.

14a) Dates defining the periods of recruitment and follow-up

Does your paper address CONSORT subitem 14a? *

Copy and paste relevant sections from the manuscript (include quotes in quotation marks "like this" to indicate direct quotes from your manuscript), or elaborate on this item by providing additional information not in the ms, or briefly explain why the item is not applicable/relevant for your study

Yes.
. Baseline assessments were completed between August 2015 and November 2016 (when the desired sample size was reached), and 12-month follow-up data collection concluded in December 2017."

14a-i) Indicate if critical “secular events” fell into the study period

Indicate if critical “secular events” fell into the study period, e.g., significant changes in Internet resources available or “changes in computer hardware or Internet delivery resources”

	1	2	3	4	5	
subitem not at all important	○	○	◉	○	○	essential

Does your paper address subitem 14a-i?

Copy and paste relevant sections from the manuscript (include quotes in quotation marks "like this" to indicate direct quotes from your manuscript), or elaborate on this item by providing additional information not in the ms, or briefly explain why the item is not applicable/relevant for your study

Not applicable. There were no critical or secular events during the study period.

14b) Why the trial ended or was stopped (early)

Does your paper address CONSORT subitem 14b? *

Copy and paste relevant sections from the manuscript (include quotes in quotation marks "like this" to indicate direct quotes from your manuscript), or elaborate on this item by providing additional information not in the ms, or briefly explain why the item is not applicable/relevant for your study

Not applicable, the trial was not stopped early.

15) A table showing baseline demographic and clinical characteristics for each group

NPT: When applicable, a description of care providers (case volume, qualification, expertise, etc.) and centers (volume) in each group

Does your paper address CONSORT subitem 15? *

Copy and paste relevant sections from the manuscript (include quotes in quotation marks "like this" to indicate direct quotes from your manuscript), or elaborate on this item by providing additional information not in the ms, or briefly explain why the item is not applicable/relevant for your study

Yes.
"The sample's characteristics have been reported in detail elsewhere [32], and included in Multimedia Appendix 2A. Parent participants were predominantly female (87.2%), married or in a de facto relationship (76.6%), employed full- or part-time (86.6%), spoke English as their primary language at home (84.1%), from an intact family situation (70.5%), and had tertiary level education (58.2%). Parents had a mean age of 45.15 years (SD=5.20) and their adolescents (55.4% male) had a mean age of 13.68 years (SD=1.06). 59.1% of parents reported having a current or past history of mental illness, whereas less than a quarter of adolescents were reported by their parents to have a current (18.9%) or past (15.9%) mental health diagnosis. There were no group differences on participant characteristics or outcome measures at baseline [32; see Multimedia Appendix 2]."

15-i) Report demographics associated with digital divide issues

In ehealth trials it is particularly important to report demographics associated with digital divide issues, such as age, education, gender, social-economic status, computer/Internet/ehealth literacy of the participants, if known.

	1	2	3	4	5	
subitem not at all important	○	○	◉	○	○	essential

Does your paper address subitem 15-i? *

Copy and paste relevant sections from the manuscript (include quotes in quotation marks "like this" to indicate direct quotes from your manuscript), or elaborate on this item by providing additional information not in the ms, or briefly explain why the item is not applicable/relevant for your study

Yes.
The demographic characteristics table includes age, gender, and education level of participants.

16) For each group, number of participants (denominator) included in each analysis and whether the analysis was by original assigned groups

16-i) Report multiple “denominators” and provide definitions

Report multiple “denominators” and provide definitions: Report N’s (and effect sizes) “across a range of study participation [and use] thresholds” [1], e.g., N exposed, N consented, N used more than x times, N used more than y weeks, N participants “used” the intervention/comparator at specific pre-defined time points of interest (in absolute and relative numbers per group). Always clearly define “use” of the intervention.

	1	2	3	4	5	
subitem not at all important	○	○	○	◉	○	essential

Does your paper address subitem 16-i? *

Copy and paste relevant sections from the manuscript (include quotes in quotation marks "like this" to indicate direct quotes from your manuscript), or elaborate on this item by providing additional information not in the ms, or briefly explain why the item is not applicable/relevant for your study

Yes. See sections "Attrition", "Intervention completion and adherence" and the participant flow diagram.

16-ii) Primary analysis should be intent-to-treat

Primary analysis should be intent-to-treat, secondary analyses could include comparing only “users”, with the appropriate caveats that this is no longer a randomized sample (see 18-i).

	1	2	3	4	5	
subitem not at all important	○	○	○	○	◉	essential

Does your paper address subitem 16-ii?

Copy and paste relevant sections from the manuscript (include quotes in quotation marks "like this" to indicate direct quotes from your manuscript), or elaborate on this item by providing additional information not in the ms, or briefly explain why the item is not applicable/relevant for your study

Yes.
"Primary and secondary outcome analyses were conducted on an intention-to-treat basis, using Mixed Model Repeated Measures (MMRM), with an unstructured covariance matrix. MMRM uses all available data from all participants, including those who withdrew from follow-up assessments [46]."

17a) For each primary and secondary outcome, results for each group, and the estimated effect size and its precision (such as 95% confidence interval)

Does your paper address CONSORT subitem 17a? *

Copy and paste relevant sections from the manuscript (include quotes in quotation marks "like this" to indicate direct quotes from your manuscript), or elaborate on this item by providing additional information not in the ms, or briefly explain why the item is not applicable/relevant for your study

Yes. All results include Cohen's D (effect size) with 95% confidence intervals.
See Tables 2, Figures 2, 3, and appendices.

17a-i) Presentation of process outcomes such as metrics of use and intensity of use

In addition to primary/secondary (clinical) outcomes, the presentation of process outcomes such as metrics of use and intensity of use (dose, exposure) and their operational definitions is critical. This does not only refer to metrics of attrition (13-b) (often a binary variable), but also to more continuous exposure metrics such as “average session length”. These must be accompanied by a technical description how a metric like a “session” is defined (e.g., timeout after idle time) [1] (report under item 6a).

	1	2	3	4	5	
subitem not at all important	○	○	○	○	○	essential

Does your paper address subitem 17a-i?

Copy and paste relevant sections from the manuscript (include quotes in quotation marks "like this" to indicate direct quotes from your manuscript), or elaborate on this item by providing additional information not in the ms, or briefly explain why the item is not applicable/relevant for your study

Yes.
"Intervention completion and adherence
As reported previously, the mean intended program use by the intervention group (n = 179) was 6.85 out of a possible nine modules. The mean observed usage by the intervention group was 5.17 modules (Yap et al., 2018). At the time of data extraction for this paper, parents in the intervention group had completed an average of 73.7% of their selected program. Intervention adherence in the intervention group was 44.1% (n = 79 parents whose observed usage equalled their intended usage). During the follow-up period (from 3 to 12-months post-baseline), 12 of the 179 intervention-group parents (6.7%) accessed a mean of 2 modules (range 1-8, SD = 2). In the control group, 33 of the 180 parents (18.3%) accessed a mean of 2 factsheets (range 1-4, SD = 1.20)."

17b) For binary outcomes, presentation of both absolute and relative effect sizes is recommended

Does your paper address CONSORT subitem 17b? *

Copy and paste relevant sections from the manuscript (include quotes in quotation marks "like this" to indicate direct quotes from your manuscript), or elaborate on this item by providing additional information not in the ms, or briefly explain why the item is not applicable/relevant for your study

Not applicable, no binary outcomes included.

18) Results of any other analyses performed, including subgroup analyses and adjusted analyses, distinguishing pre-specified from exploratory

Does your paper address CONSORT subitem 18? *

Copy and paste relevant sections from the manuscript (include quotes in quotation marks "like this" to indicate direct quotes from your manuscript), or elaborate on this item by providing additional information not in the ms, or briefly explain why the item is not applicable/relevant for your study

Yes. See results sections:
"Mediation Analyses" and "Post Hoc Moderation Analyses"

18-i) Subgroup analysis of comparing only users

A subgroup analysis of comparing only users is not uncommon in ehealth trials, but if done, it must be stressed that this is a self-selected sample and no longer an unbiased sample from a randomized trial (see 16-iii).

	1	2	3	4	5	
subitem not at all important	○	○	◉	○	○	essential

Does your paper address subitem 18-i?

Copy and paste relevant sections from the manuscript (include quotes in quotation marks "like this" to indicate direct quotes from your manuscript), or elaborate on this item by providing additional information not in the ms, or briefly explain why the item is not applicable/relevant for your study

Not applicable, we did not conduct subgroup analyses of users only.

19) All important harms or unintended effects in each group

(for specific guidance see CONSORT for harms)

Does your paper address CONSORT subitem 19? *

Copy and paste relevant sections from the manuscript (include quotes in quotation marks "like this" to indicate direct quotes from your manuscript), or elaborate on this item by providing additional information not in the ms, or briefly explain why the item is not applicable/relevant for your study

Not applicable, there were no harms or unintended effects.

19-i) Include privacy breaches, technical problems

Include privacy breaches, technical problems. This does not only include physical “harm” to participants, but also incidents such as perceived or real privacy breaches [1], technical problems, and other unexpected/unintended incidents. “Unintended effects” also includes unintended positive effects [2].

	1	2	3	4	5	
subitem not at all important	○	○	◉	○	○	essential

Does your paper address subitem 19-i?

Copy and paste relevant sections from the manuscript (include quotes in quotation marks "like this" to indicate direct quotes from your manuscript), or elaborate on this item by providing additional information not in the ms, or briefly explain why the item is not applicable/relevant for your study

Not applicable, there were no privacy breaches or technical problems.

19-ii) Include qualitative feedback from participants or observations from staff/researchers

Include qualitative feedback from participants or observations from staff/researchers, if available, on strengths and shortcomings of the application, especially if they point to unintended/unexpected effects or uses. This includes (if available) reasons for why people did or did not use the application as intended by the developers.

	1	2	3	4	5	
subitem not at all important	○	○	◉	○	○	essential

Does your paper address subitem 19-ii?

Copy and paste relevant sections from the manuscript (include quotes in quotation marks "like this" to indicate direct quotes from your manuscript), or elaborate on this item by providing additional information not in the ms, or briefly explain why the item is not applicable/relevant for your study

Not applicable, we did not collect qualitative feedback.

DISCUSSION

22) Interpretation consistent with results, balancing benefits and harms, and considering other relevant evidence

NPT: In addition, take into account the choice of the comparator, lack of or partial blinding, and unequal expertise of care providers or centers in each group

22-i) Restate study questions and summarize the answers suggested by the data, starting with primary outcomes and process outcomes (use)

Restate study questions and summarize the answers suggested by the data, starting with primary outcomes and process outcomes (use).

	1	2	3	4	5	
subitem not at all important	○	○	○	◉	○	essential

Does your paper address subitem 22-i? *

Copy and paste relevant sections from the manuscript (include quotes in quotation marks "like this" to indicate direct quotes from your manuscript), or elaborate on this item by providing additional information not in the ms, or briefly explain why the item is not applicable/relevant for your study

Yes.
See Discussion sections: "Principle findings: Parent-report of parenting", "Principle findings: Adolescent-report of parenting" and "Principle findings: Adolescent symptoms"

22-ii) Highlight unanswered new questions, suggest future research

Highlight unanswered new questions, suggest future research.

	1	2	3	4	5	
subitem not at all important	○	○	○	◉	○	essential

Does your paper address subitem 22-ii?

Copy and paste relevant sections from the manuscript (include quotes in quotation marks "like this" to indicate direct quotes from your manuscript), or elaborate on this item by providing additional information not in the ms, or briefly explain why the item is not applicable/relevant for your study

Yes. This is included throughout the Discussion section. In particular, see sections:
"Comparison with prior work" and "strengths and limitations".

20) Trial limitations, addressing sources of potential bias, imprecision, and, if relevant, multiplicity of analyses

20-i) Typical limitations in ehealth trials

Typical limitations in ehealth trials: Participants in ehealth trials are rarely blinded. Ehealth trials often look at a multiplicity of outcomes, increasing risk for a Type I error. Discuss biases due to non-use of the intervention/usability issues, biases through informed consent procedures, unexpected events.

	1	2	3	4	5	
subitem not at all important	○	○	○	◉	○	essential

Does your paper address subitem 20-i? *

Copy and paste relevant sections from the manuscript (include quotes in quotation marks "like this" to indicate direct quotes from your manuscript), or elaborate on this item by providing additional information not in the ms, or briefly explain why the item is not applicable/relevant for your study

Yes. See Discussion section "Strengths and limitations".

21) Generalisability (external validity, applicability) of the trial findings

NPT: External validity of the trial findings according to the intervention, comparators, patients, and care providers or centers involved in the trial

21-i) Generalizability to other populations

Generalizability to other populations: In particular, discuss generalizability to a general Internet population, outside of a RCT setting, and general patient population, including applicability of the study results for other organizations

	1	2	3	4	5	
subitem not at all important	○	○	○	◉	○	essential

Does your paper address subitem 21-i?

Copy and paste relevant sections from the manuscript (include quotes in quotation marks "like this" to indicate direct quotes from your manuscript), or elaborate on this item by providing additional information not in the ms, or briefly explain why the item is not applicable/relevant for your study

Yes. See Discussion section "Strengths and limitations".

21-ii) Discuss if there were elements in the RCT that would be different in a routine application setting

Discuss if there were elements in the RCT that would be different in a routine application setting (e.g., prompts/reminders, more human involvement, training sessions or other co-interventions) and what impact the omission of these elements could have on use, adoption, or outcomes if the intervention is applied outside of a RCT setting.

	1	2	3	4	5	
subitem not at all important	○	○	◉	○	○	essential

Does your paper address subitem 21-ii?

Copy and paste relevant sections from the manuscript (include quotes in quotation marks "like this" to indicate direct quotes from your manuscript), or elaborate on this item by providing additional information not in the ms, or briefly explain why the item is not applicable/relevant for your study

No.

OTHER INFORMATION

23) Registration number and name of trial registry

Does your paper address CONSORT subitem 23? *

Copy and paste relevant sections from the manuscript (include quotes in quotation marks "like this" to indicate direct quotes from your manuscript), or elaborate on this item by providing additional information not in the ms, or briefly explain why the item is not applicable/relevant for your study

Yes.
"The trial was prospectively registered with the Australian New Zealand Clinical Trials Registry (registration number ANZCTR12615000328572)"

24) Where the full trial protocol can be accessed, if available

Does your paper address CONSORT subitem 24? *

Cite a Multimedia Appendix, other reference, or copy and paste relevant sections from the manuscript (include quotes in quotation marks "like this" to indicate direct quotes from your manuscript), or elaborate on this item by providing additional information not in the ms, or briefly explain why the item is not applicable/relevant for your study

The trial registration can be accessed via the Australian New Zealand Clinical Trials Registry, with the registration number provided in the paper.

25) Sources of funding and other support (such as supply of drugs), role of funders

Does your paper address CONSORT subitem 25? *

Copy and paste relevant sections from the manuscript (include quotes in quotation marks "like this" to indicate direct quotes from your manuscript), or elaborate on this item by providing additional information not in the ms, or briefly explain why the item is not applicable/relevant for your study

Yes.
"Acknowledgments
The authors acknowledge funding from the National Health and Medical Research Council (NHMRC) for the web development of the PiP intervention, and the partnership of beyondblue, the national depression and anxiety initiative in the development of the parenting guidelines. The authors received salary support from the NHMRC for a Career Development Fellowship (MBHY, APP1061744) and a Senior Principal Research Fellowship (AFJ, APP1059785), an Australian Research Council Laureate Fellowship (RMR, FL150100096), and a Monash University Postgraduate Publication Award (MCB). The RCT was supported by an Australian Rotary Health Research Grant and Monash University Advancing Women's Research Success Grant. None of the funding sources had any role in the conduct of publication of this study."

X27) Conflicts of Interest (not a CONSORT item)

X27-i) State the relation of the study team towards the system being evaluated

In addition to the usual declaration of interests (financial or otherwise), also state the relation of the study team towards the system being evaluated, i.e., state if the authors/evaluators are distinct from or identical with the developers/sponsors of the intervention.

	1	2	3	4	5	
subitem not at all important	○	○	○	●	○	essential

Does your paper address subitem X27-i?

Copy and paste relevant sections from the manuscript (include quotes in quotation marks "like this" to indicate direct quotes from your manuscript), or elaborate on this item by providing additional information not in the ms, or briefly explain why the item is not applicable/relevant for your study

Yes.
"Conflicts of interest
MBHY, MCB, AFJ and KL are co-developers of the PiP intervention, and MBHY and AFJ are cofounders of the broader Parenting Strategies online platform of parenting interventions, including PiP. None of the authors derive any personal financial benefit from these interventions."

About the CONSORT EHEALTH checklist

As a result of using this checklist, did you make changes in your manuscript? *

- ( ) yes, major changes
- (•) yes, minor changes
- ( ) no

What were the most important changes you made as a result of using this checklist?

Your answer

How much time did you spend on going through the checklist INCLUDING making changes in your manuscript *

4hrs

As a result of using this checklist, do you think your manuscript has improved? *

- (x) yes
- ( ) no
- ( ) Other:

Would you like to become involved in the CONSORT EHEALTH group?

This would involve for example becoming involved in participating in a workshop and writing an "Explanation and Elaboration" document

- ( ) yes
- (x) no
- ( ) Other:

Any other comments or questions on CONSORT EHEALTH

Your answer

STOP - Save this form as PDF before you click submit

To generate a record that you filled in this form, we recommend to generate a PDF of this page (on a Mac, simply select "print" and then select "print as PDF") before you submit it.

When you submit your (revised) paper to JMIR, please upload the PDF as supplementary file.

Don't worry if some text in the textboxes is cut off, as we still have the complete information in our database. Thank you!

Final step: Click submit !

Click submit so we have your answers in our database!

SUBMIT

Never submit passwords through Google Forms.

This content is neither created nor endorsed by Google. Report Abuse - Terms of Service

Google Forms
